# Cellular responses to a prolonged delay in mitosis are determined by a DNA damage response controlled by Bcl-2 family proteins

**DOI:** 10.1098/rsob.140156

**Published:** 2015-03-11

**Authors:** Didier J. Colin, Karolina O. Hain, Lindsey A. Allan, Paul R. Clarke

**Affiliations:** Division of Cancer Research, Medical Research Institute, University of Dundee, Jacqui Wood Cancer Centre, Ninewells Hospital and Medical School, Dundee DD1 9SY, UK

**Keywords:** mitosis, apoptosis, DNA damage response, paclitaxel, caspase

## Abstract

Anti-cancer drugs that disrupt mitosis inhibit cell proliferation and induce apoptosis, although the mechanisms of these responses are poorly understood. Here, we characterize a mitotic stress response that determines cell fate in response to microtubule poisons. We show that mitotic arrest induced by these drugs produces a temporally controlled DNA damage response (DDR) characterized by the caspase-dependent formation of γH2AX foci in non-apoptotic cells. Following exit from a delayed mitosis, this initial response results in activation of DDR protein kinases, phosphorylation of the tumour suppressor p53 and a delay in subsequent cell cycle progression. We show that this response is controlled by Mcl-1, a regulator of caspase activation that becomes degraded during mitotic arrest. Chemical inhibition of Mcl-1 and the related proteins Bcl-2 and Bcl-x_L_ by a BH3 mimetic enhances the mitotic DDR, promotes p53 activation and inhibits subsequent cell cycle progression. We also show that inhibitors of DDR protein kinases as well as BH3 mimetics promote apoptosis synergistically with taxol (paclitaxel) in a variety of cancer cell lines. Our work demonstrates the role of mitotic DNA damage responses in determining cell fate in response to microtubule poisons and BH3 mimetics, providing a rationale for anti-cancer combination chemotherapies.

## 2. Introduction

Microtubule poisons such as taxol (paclitaxel) disrupt mitotic spindle assembly and delay or arrest proliferating cells in a prometaphase-like state through the prolonged activity of the spindle assembly checkpoint (SAC), which restrains anaphase by inhibiting the widespread destruction of mitotic regulators such as cyclin B1 and securin [1]. Cells held in mitosis by microtubule poisons can undergo caspase-dependent apoptosis [2], which is influenced by the duration of the mitotic arrest [3,4], or they can slip out of mitosis after a prolonged period [5,6]. Premature exit from mitosis usually decreases sensitivity to microtubule poisons [4], consistent with the generation of apoptosis-promoting activity during mitotic arrest. Apoptosis can still occur, however, after exit from mitosis [3,6]. The balance between these cell fates is likely to determine the effectiveness of microtubule poisons and other anti-mitotic drugs in cancer chemotherapy [3,4,6–9].

Control of apoptosis during mitosis is determined by protein phosphorylation and the timed, ubiquitin-mediated destruction of key regulators of the intrinsic apoptotic pathway by the proteasome [10]. If metaphase is not successfully resolved, then apoptosis can be initiated after the anti-apoptotic protein Mcl-1 is degraded, which occurs subsequent to its phosphorylation by CDK1–cyclin B1 [11,12]. In addition, the related anti-apoptotic proteins Bcl-2 and Bcl-x_L_ are phosphorylated and might be inhibited during mitotic arrest [13]. The slow degradation of cyclin B1 even though the SAC is active can lead eventually to release from mitosis [5]. Whether or not apoptosis is initiated during the arrest or after a cell enters interphase is thought to depend upon the relative timing of changes in the activities of CDK1–cyclin B1 and regulators of apoptosis during mitotic arrest [2,6,10].

A prolonged delay in mitosis can also determine subsequent cell proliferation [14]. However, the nature of the putative mitotic stress signal and the mechanism by which delayed mitosis can influence subsequent cell cycle progression have been unclear. Recent research has shown that there is an accumulation of DNA damage in cells arrested in mitosis, as evidenced by an increase in phosphorylated histone H2AX (γH2AX) [15,16], and the transcription factor p53 is induced after release into interphase [14,15,17]. Evidence has also been provided that mitotic arrest can cause DNA damage owing to partial apoptosis [17]. These findings suggest that stress responses initiated by mitotic disruption can determine subsequent cell cycle progression and that this process might be related to the mechanism of apoptosis.

Here, we show that a prolonged delay in mitosis initiates a time-dependent DDR that controls subsequent cell fate through the activation of protein kinases and p53. This pathway is dependent on subapoptotic caspase activity and is controlled by Bcl-2 family proteins. Thus, we identify a potential novel mechanism for the oncogenic activity of Bcl-2 family proteins through indirect control of the cell cycle and apoptosis. We also demonstrate that pharmacological manipulation of Bcl-2 family proteins or DDR kinases enhances the cell-killing effects of anti-mitotic drugs on proliferating cancer cells by enhancing the response to mitotic stress, providing a rationale for combination chemotherapy.

## 3. Material and methods

### 3.1. Reagents and antibodies

Reagents were purchased from Sigma-Aldrich unless otherwise specified. The origins of primary antibodies and the dilutions used in this study are described in the electronic supplementary material, table S1. Secondary antibodies used for Western blot and immunofluorescence analyses were from Biorad and Molecular Probes, respectively.

### 3.2. Cells

U2OS (HTB96) cells were obtained from Cell Services, Cancer Research UK London Research Institute. U2OS cells in which p53 was stably depleted by shRNA (pRS-p53) and control cells transfected with a scrambled shRNA (pRS-sc) [18] were a gift from K. Ryan (Beatson Institute for Cancer Research, Glasgow, UK). All the other cell lines were purchased from ATCC (LGC Standards, Teddington, UK).

### 3.3. Cell synchronizations and treatments

U2OS cells were synchronized in mitosis by collection of rounded-up cells after a 2 h treatment with a microtubule poison and then either replated with the drug to maintain mitotic arrest or released in fresh media. Untreated mitotic cells were obtained by washing-off asynchronous cultures. Synchronization at the G1/S boundary by double thymidine block was performed as described [11]. G1 phase cells were obtained by centrifugal elutriation [19]. U2OS cells were transfected with Mcl-1 subcloned into pCMV-Flag (MRC Protein Phosphorylation Unit, Dundee) using Superfect transfection reagent (Qiagen). Mcl-1 was depleted in U2OS cells by the siRNA GGACUUUUAGAUUUAGUGA (MWG) using RNAiMax (Invitrogen) as transfection reagent. Cells were treated as indicated with z-VAD-fmk (Enzo Life Sciences), Obatoclax/GX15–070, Navitoclax/ABT-263, NVP-BEZ235 (Selleck Chemicals) or KU55933, NU7441, SB218078 (Tocris).

### 3.4. Flow cytometry

One- and two-dimensional cell cycle analyses, apoptosis determined by fluorochrome-labelled inhibitor of caspases (FLICA, FAM-DEVD-fmk, Immunochemistry Technologies) and immunofluorescence analyses by flow cytometry were performed according to manufacturers instructions or as described previously [11] and detailed in the electronic supplementary material.

### 3.5. Western blot analyses

Protein extracts were prepared using a standard RIPA protocol and analysed as described in the electronic supplementary material.

### 3.6. Proliferation assays

Cell viability was monitored in an IncuCyte ZOOM (Essen) according to the manufacturer's instructions or by a standard crystal violet assay (see the electronic supplementary material for details). Synergies between drugs were determined according to the Chou–Talalay method [20] by using a crystal violet-based assay detailed in the electronic supplementary material.

### 3.7. Immunofluorescence assays and microscopy

γH2AX immuno-detection was performed according to a standard protocol detailed in the electronic supplementary material.

### 3.8. Figures and statistics

Statistical analyses and regressions were conducted using Graphpad Prism. Differences between samples were assessed using non-parametric Mann–Whitney or one-way ANOVA statistical tests as required. Figures were generated using Microsoft Excel, Graphpad Prism, Adobe Photoshop and Adobe Illustrator.

## 4. Results

### 4.1. Mitotic delay induced by microtubule poisons activates DNA damage signalling and affects subsequent cell cycle progression and apoptosis

**Figure 1.**
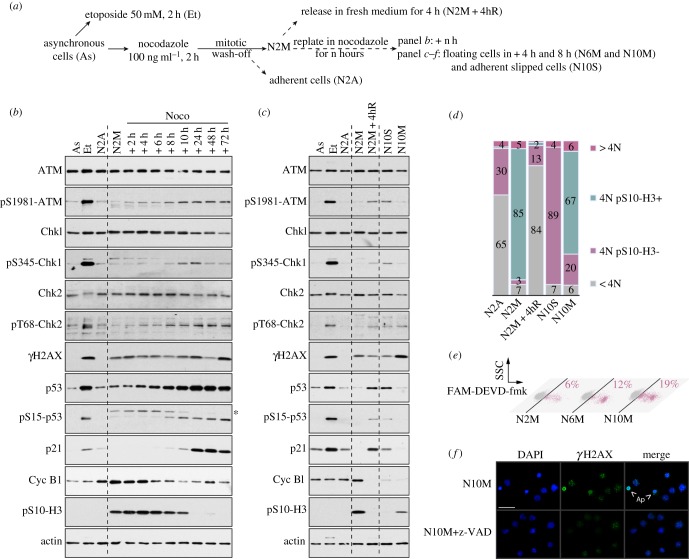
The period of mitotic arrest controls subsequent DNA damage signalling, cell cycle progression and apoptosis. (*a*) Experimental protocol. (*b*) DNA damage signalling in response to a prolonged mitotic arrest. Samples were analysed by immunoblotting using the antibodies indicated. Asterisk denotes a non-specific signal on pS15-p53 blot. (*c,d*) DNA damage signalling in G1 following mitotic arrest. Samples were analysed by immunoblotting using specified antibodies (*c*), and cell cycle profiles were characterized by flow cytometry using propidium iodide as a marker of DNA content and phosphorylated Ser10 histone H3 as a marker of mitosis (*d*). (*e*) Induction of apoptosis during a mitotic arrest. Cells were incubated with a FAM-DEVD-fmk probe and analysed by flow cytometry. Percentages represent the amount of active cleaved caspase-3/7 positive cells. (*f*) γH2AX induction in cells arrested in mitosis is caspase-dependent. Cells were synchronized for 10 h in mitosis in the presence of z-VAD-fmk where indicated, cytospun and immunostained using an anti-γH2AX antibody. Representative microscopic fields are shown; Ap, apoptotic cells; scale bar, 50 µm.

To study cellular responses to mitotic arrest, we used a cell culture model in which rounded-up mitotic human osteosarcoma cells (U2OS) were collected after treatment of an asynchronous culture with nocodazole for 2 h (denoted N2M; figure 1*a*; electronic supplementary material, figure S1A). Mitotic cells were then replated in nocodazole for a further period and analysed by Western blotting (figure 1*b*). This procedure allows analysis of biochemical changes during the period of mitotic arrest and after subsequent entry into interphase without interference from cellular stresses generated by other methods of pre-synchronization. We found that a prolonged mitotic delay of up to 10 h followed by slippage into interphase (confirmed by flow cytometry; electronic supplementary material, figure S1B) activated components of the DNA damage response (DDR) [21] in a time-dependent manner (figure 1*b*). Ataxia–telangiectasia-mutated (ATM), a large phosphatidylinositol-3-kinase-related protein kinase (PIKK), became phosphorylated at Ser1981, a site associated with its activation and stabilization at sites of DNA damage [22]. The phosphorylation of ATM increased and persisted as cells slipped out of mitosis after 10 h (indicated by the loss of cyclin B1 and histone H3 Ser10 dephosphorylation), and correlated with activating phosphorylation of its targets, the protein kinase Chk2 and p53; there was also a transient activating phosphorylation of Chk1 at Ser345, a site targeted by ATR (ATM- and Rad3-related).

We confirmed that histone H2AX phosphorylated on Ser139 (γH2AX), a site targeted by ATM and the related kinases ATR- and DNA-dependent protein kinase (DNA-PK), was increased during mitotic arrest and elevated further after slippage into interphase. We also observed a strong increase upon exit from mitosis of the protein levels of both p53 and its transcriptional target, the p21^waf1^ cyclin-dependent kinase inhibitor (figure 1*b*). Together, these results confirm that a prolonged delay in mitosis initiates a primary DDR that is fulfilled in the subsequent interphase after cells have slipped out of mitosis.

We also analysed cells released from a transient mitotic arrest by washing out nocodazole after 2 h of treatment (figure 1*c*). Released cells (denoted N2M + 4hR) exhibited activation of the DDR in a similar fashion to cells that had slipped out of mitosis despite the continued presence of the drug (N10S). Flow cytometry confirmed that released cells underwent cytokinesis to produce diploid G1 cells with a 2N DNA content, whereas slipped cells gave rise to a tetraploid (4N) G1 population owing to failure to undergo cytokinesis (figure 1*d*). Thus, the secondary DDR induced after a prolonged delay in mitosis is not dependent on whether or not cells exit mitosis aberrantly and become tetraploid. In the corresponding population of floating cells that had not slipped out of mitosis (N10M; figure 1*c*), there was a strong increase in γH2AX that is likely to be due to widespread DNA fragmentation in apoptotic cells that accumulated during mitotic arrest (figure 1*e*). The caspase inhibitor zVAD-fmk blocked the generation of such apoptotic cells as expected (figure 1*f*). This is consistent with the ability of some mitotically arrested cells to proceed into apoptosis without first entering interphase [3,6].

In contrast to G1 cells previously arrested in mitosis, elutriated G1 cells did not exhibit any induction of the DDR (electronic supplementary material, figure S1E). Furthermore, we did not detect any effect of nocodazole or taxol on the phosphorylation or protein levels of ATM, Chk2, γH2AX, p53 or p21^waf1^ in cells treated only during interphase (electronic supplementary material, figure S2A,C). Irrespective of the effects of the microtubule poisons, we observed a transient increase in the activating phosphorylation of Chk1 in interphase cells progressing through G1 into S-phase, consistent with a role for this kinase in the normal control of DNA replication (electronic supplementary material, figure S2B,D). We also found that interphase cells synchronized by a double thymidine block exhibited a significant induction of γH2AX (electronic supplementary material, figure S2C; 0 h samples), which is attributable to DNA replication stress. Because some of this damage might persist into mitosis, we avoided the further use of this protocol to study mitotic responses.

### 4.2. The period of mitotic arrest controls DNA damage signalling, cell cycle progression and apoptosis

**Figure 2.**
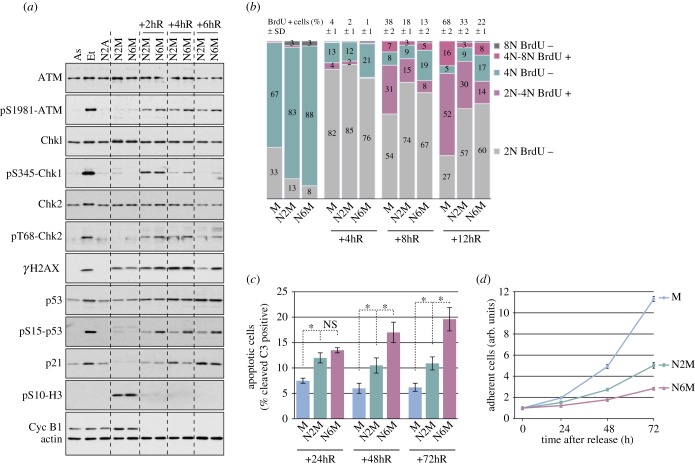
The period of mitotic arrest determines subsequent cell fate. (*a*) The period of mitotic arrest controls subsequent DNA damage signalling. U2OS cells were arrested in mitosis for 2 (N2M) or 6 h (N6M) prior to release from mitosis for 2, 4 or 6 h and analysed by immunoblotting using the specified antibodies. Asynchronous cells (As), etoposide-treated cells (Et) and cells remaining adherent after 2 h treatment with nocodazole (N2A) were also analysed for comparison. (*b*) The period of mitotic arrest controls subsequent cell cycle progression. U2OS cells were treated as in (*a*) and untreated control mitotic cells were used as controls (M). BrdU incorporation to mark newly synthesized DNA was analysed by flow cytometry. Cumulative histograms show the percentages of cells in different phases of the cell cycle, according to the BrdU incorporation and DNA content. On top, percentages of BrdU positive cells (S phase cells) are given as means ± s.d. (*n* ≥ 3). (*c,d*) The period of mitotic arrest controls subsequent cell viability and proliferation. Cells treated as in (*a*) were collected at indicated times, incubated with an FAM-DEVD-fmk fluorescent probe for apoptotic caspase 3/7 activity and analysed by flow cytometry (*c*); values are means ± s.d. (*n* ≥ 3). Statistical differences were analysed with the Mann–Whitney test; n.s., non-significant, *p < 0.05. The relative numbers of viable, adherent cells were determined by crystal violet assay at indicated times (*d*). Values are means ± s.d. from quadruplicate wells of a representative experiment (*n* ≥ 3).

To test whether the period of mitotic arrest affects subsequent responses, we maintained cells for either 2 or 6 h in nocodazole-induced mitotic arrest, then released the cells by washing out the drug and analysed the DDR. Cells that had been arrested in mitosis for a total of 6 h exhibited stronger activating phosphorylation of ATM, Chk2 and p53 after release from mitosis and they were more likely to be delayed in G1 phase (BrdU negative adherent cells with 2N DNA content) than those that had been arrested in mitosis for only 2 h (figure 2*a,b*). The activating phosphorylation of Chk1 was also prolonged (figure 2*a*), consistent with a delay in S phase initiation (figure 2*b*). Similar results were obtained when cells were treated with taxol rather than treated with nocodazole (electronic supplementary material, figure S3A,B). Thus, the period of mitotic arrest determines the intensity of the DNA damage signalling and this correlates with restraint of subsequent cell cycle progression. Moreover, we found that U2OS cells arrested for longer in mitosis died more readily by apoptosis after release into interphase (figure 2*c* and electronic supplementary material, figure S3C). Together, these effects on cell cycle progression and apoptosis resulted in the strong inhibition of cell proliferation (figure 2*d*; electronic supplementary material, figure S3D).

### 4.3. The DNA damage response in mitotic cells is caspase-dependent and is regulated by Bcl-2 family proteins

**Figure 3.**
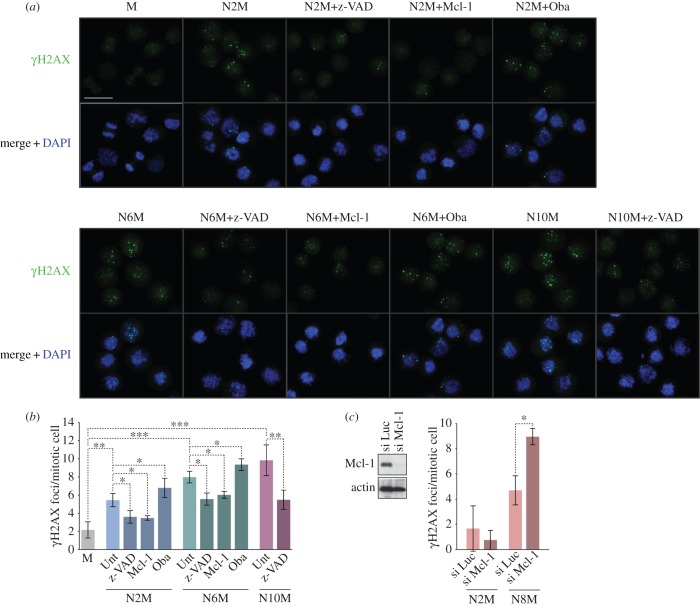
Mitotic arrest elicits a localized caspase-dependent DNA damage response under the control of Bcl-2 family proteins. Cells were synchronized in prolonged mitosis for different times (N2M, N6M, N10M) and compared with untreated mitotic cells (M). Some cells were co-treated with the pan-caspase inhibitor z-VAD-fmk (20 µM) or the Mcl-1/Bcl-2/Bcl-x_L_ inhibitor Obatoclax (500 nM). U2OS cells over-expressing Mcl-1 were prepared by transient transfection. Cells were cytospun and immunostained using an anti-γH2AX antibody. (*a*) Representative microscopic fields are shown; scale bar, 40 µm. (*b,c*) The histograms show the numbers of γH2AX foci per mitotic cell treated as indicated in (*a*) or in which Mcl-1 was depleted by siRNA (si Mcl-1) compared with control cells in which an irrelevant luciferase siRNA was transfected (si Luc); values are means ± s.d. (*n* ≥ 3). Statistical differences were analysed using one-way ANOVA statistical tests; *p < 0.05, **p < 0.01 and ***p < 0.001.

In agreement with previous descriptions [15,16], we found that most cells arrested in mitosis for 2 or more hours exhibited localized γH2AX foci (figure 3). Counting the number of γH2AX foci, we found that untreated mitotic cells exhibited only occasional foci (figure 3*a*) with a mean of 2.2 foci per cell (figure 3*b*). By contrast, cells arrested in mitosis had a distribution of up to 20 foci per cell (electronic supplementary material, figure S4), with a mean of 5.4 in cells arrested for 2 h. The mean number of foci per cell increased when the period of the arrest was prolonged, showing that it is time-dependent (figure 3*b*; electronic supplementary material, figure S4). Importantly, we found that the formation of these foci was inhibited by the caspase inhibitor zVAD-fmk (figure 3). By contrast, γH2AX foci induced in mitotic cells by the topoisomerase II poison etoposide, which causes widespread DNA double-strand breaks, were not dependent on caspase activity (electronic supplementary material, figure S5).

Mcl-1 is gradually degraded during a prolonged mitotic arrest [11,12], but ectopic expression of Mcl-1 inhibited the formation of γH2AX foci in cells arrested in mitosis. Conversely, the Mcl-1/Bcl-2/Bcl-x_L_ inhibitor Obatoclax (GX15-070) [23] (figure 3*b*; electronic supplementary material, S4B) or siRNA-mediated ablation of Mcl-1 expression (figure 3*c*) increased the number of γH2AX foci per cell. These results demonstrate that many of the γH2AX foci formed during mitotic arrest require caspase activity and are under the control of Mcl-1, and possibly other related regulators of caspase activation. The distribution of the number of foci per cell (electronic supplementary material, figure S4B) suggests a stochastic element in the mechanism initiating foci damage.

### 4.4. Mcl-1 controls the DNA damage response and p53 activation following a delayed mitosis

**Figure 4.**
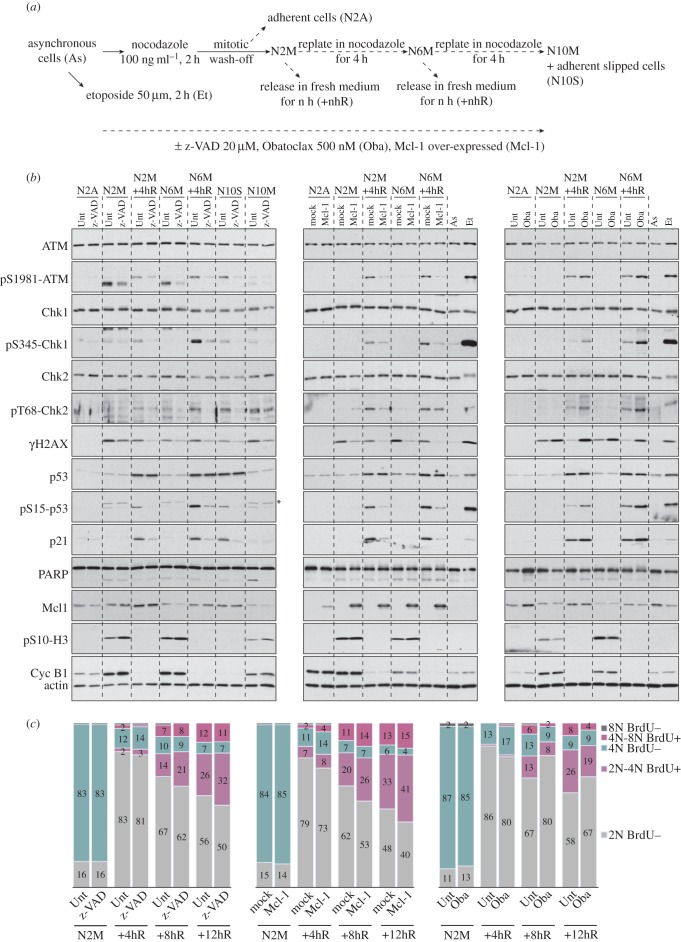
Mcl-1 controls caspase-dependent DNA damage signalling and subsequent fate of cells following mitotic arrest. (*a*) Experimental protocol. (*b*) Regulation of caspase activity affects DNA damage signalling in response to mitotic arrest. Where indicated, mitotically arrested U2OS cells were co-treated with *z*-VAD-fmk (left panel) or Obatoclax (right panel). U2OS cells expressing exogenous Mcl-1 were compared with mock-transfected controls (middle panel). Samples were analysed by immunoblotting using the specified antibodies; asterisk denotes a non-specific signal on pS15-p53 blot. (*c*) Regulation of caspase activity affects cell cycle progression following a mitotic arrest. Cells were treated as indicated in (*a*) and analysed by flow cytometry. The cumulative histograms show the percentages of cells in the different phases of the cell cycle, according to BrdU incorporation into newly synthesized DNA and DNA content by propidium iodide staining. Percentages shown are from a representative experiment (*n* ≥ 3).

Analysis of the DDR in interphase cells following release from mitotic arrest (figure 4*a*) showed that the activating phosphorylation of ATM, Chk1, Chk2 and p53, as well as the induction of p21, were strongly inhibited by addition of zVAD-fmk during the arrest (figure 4*b*, left panel), and were therefore dependent on upstream caspase activity. This is consistent with a mechanism in which the response to caspase-dependent DNA damage foci generated during a delayed mitosis is enhanced following release into interphase. By contrast, the increase in total p53 protein following mitotic delay was not affected by zVAD-fmk, indicative of a caspase-independent mechanism for p53 induction. Caspase-dependent cleavage of poly(ADP ribose) polymerase (PARP) was not observed until 10 h of incubation (N10M), consistent with flow cytometry data (figure 1*e*) showing that, until this time, few cells were undergoing apoptosis. Remarkably, however, treatment with zVAD-fmk reduced the delay in G1 of non-apoptotic cells released from 2 h mitotic arrest and promoted their entry into S phase (figure 4*c*, left panel).

The amount of Mcl-1 was diminished more markedly by 6 h mitotic arrest than by 2 h mitotic arrest, and this loss was not affected by zVAD-fmk (figure 4*b*, left panel), in agreement with the caspase-independent degradation of this protein in an APC/C-dependent manner during mitotic arrest [11]. Ectopic expression of Mcl-1 strongly inhibited the DDR (figure 4*b*, middle panel) and also promoted subsequent entry into S phase of cells that had been delayed in mitosis (figure 4*c*, middle panel). Conversely, inhibition of Mcl-1 and the related anti-apoptotic proteins Bcl-2 and Bcl-x_L_ by Obatoclax enhanced the DDR (figure 4*b*, right panel) and delayed cells in G1 (figure 4*c*, right panel), whereas no significant apoptotic cleavage of PARP was detected (figure 4*b*, right panel). Neither ectopic Mcl-1 expression nor Obatoclax treatment affected the induction of p53 protein following mitotic arrest, but they did strongly inhibit or enhance, respectively, the phosphorylation of p53 at the activating site serine 15, and there was a corresponding effect on the induction of p21. Thus, activation of the p53 pathway by DNA damage signalling after mitotic delay, with the subsequent regulation of cell cycle progression, requires caspase activity and is under the control of Mcl-1, and possibly other Bcl-2 family proteins.

### 4.5. The role of p53 in the fate of cells following mitotic delay

**Figure 5.**
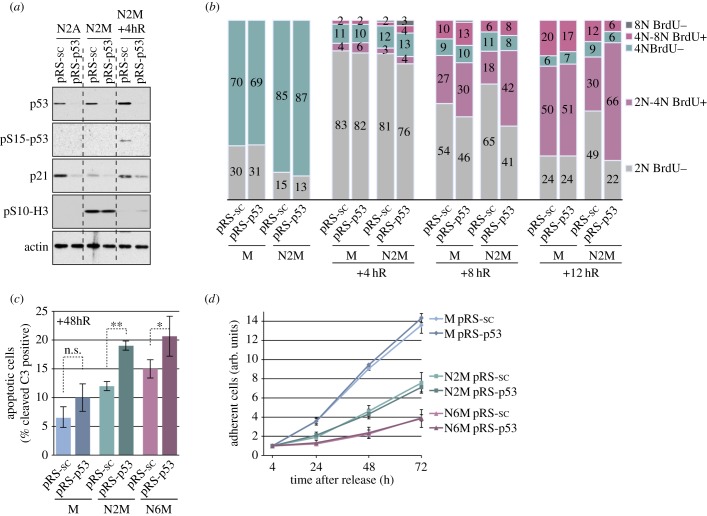
The role of p53 in the fate of cells following a mitotic arrest. (*a*) Characterization of p53-depleted U2OS cells. Stably transfected U2OS cells were treated as in figure 1*a* and analysed by immunoblotting using the specified antibodies. (*b*) Role of p53 in cell cycle progression following a mitotic arrest. Stably transfected U2OS cells treated as indicated were analysed by flow cytometry. The cumulative histograms show the percentage of cells in the different phases of the cell cycle, according to BrdU incorporation and DNA content. Data shown are from a representative experiment repeated three times. (*c,d*) Role of p53 in the viability and proliferation of cells following a mitotic arrest. (*c*) Cells treated as depicted in (*a*) were incubated with an FAM-DEVD-fmk probe and analysed by flow cytometry. The percentage of apoptotic cells with active caspase 3/7 is shown, values are means ± s.d. (*n* = 3). Statistical differences were analysed with the Mann–Whitney test; n.s., non-significant, *p < 0.05, **p < 0.01. (*d*) The relative number of viable, adherent cells was determined by following cell proliferation using IncuCyte-based cell density analyses. Values are means ± s.d. from quadruplicate wells of a representative experiment repeated three times.

Because mitotic delay resulted in the activation of p53 downstream of DNA damage signalling, we tested the role of p53 in the cellular response to a synchronized mitotic delay (figure 5). We examined the effects of mitotic arrest on a cell line derived from U2OS cells [18] in which p53 was stably depleted by expression of an shRNA (pRS-p53); cells expressing a scrambled shRNA (pRS-sc) were used for comparison. Depletion of p53 inhibited the induction of p21 following arrest in mitosis by nocodazole for 2 h (figure 5*a*) and prevented the accumulation of cells in the subsequent G1 phase (figure 5*b*). By contrast, loss of p53 did not affect the normal cell cycle progression of mitotic cells collected without nocodazole treatment. Interestingly, the rate of progression into S-phase (2N-4N BrdU positive cells) was apparently enhanced in p53-depleted cells by prior mitotic delay as compared with those cells that had progressed from a normal mitosis (figure 5*b*). These results demonstrate that p53 plays a key role in restraining cell cycle progression following mitotic stress.

Depletion of p53 also caused a significant increase in apoptotic caspase-3 activity measured 48 h after release from mitotic arrest (figure 5*c*). p53 therefore has an anti-apoptotic, pro-survival function following mitotic stress. Nevertheless, depletion of p53 had no overall effect on the number of cells that accumulated after release from mitosis, even though the effect of period of mitotic arrest was apparent (figure 5*d*). This suggests that, in this cell type, the lack of restraint on cell cycle progression in the absence of p53 is counteracted by an increase in apoptosis, so that overall cell proliferation is inhibited to the same extent by mitotic stress whether or not the cells can activate p53. Qualitatively similar results were obtained in experiments using either nocodazole (figure 5) or taxol (electronic supplementary material, figure S6) to induce mitotic arrest, although cells arrested using taxol were more prone to subsequent apoptosis or inhibition of cell proliferation than cells arrested for the same period with nocodazole (electronic supplementary material, figures S3 and S6).

### 4.6. Inhibition of DNA damage response kinases enhances the effects of mitotic stress

**Figure 6.**
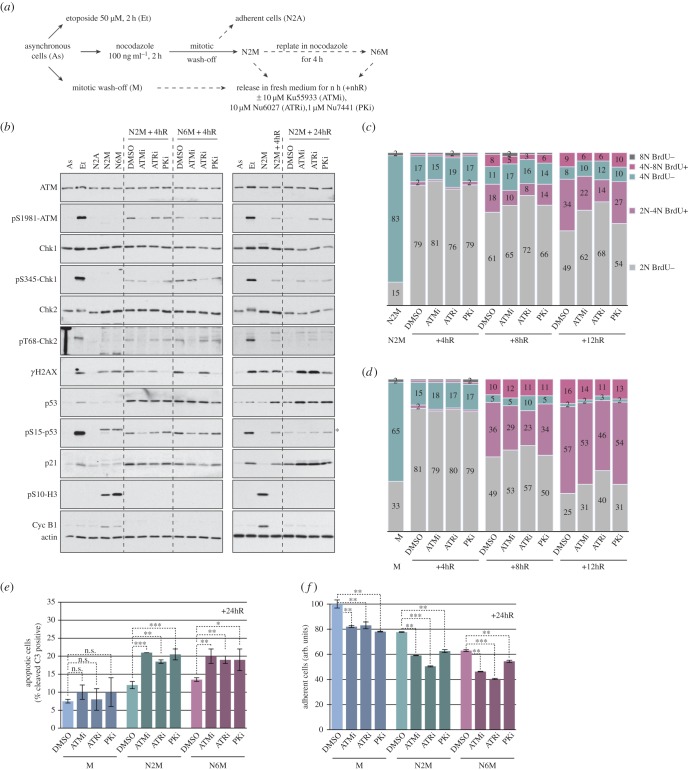
Inhibition of DNA damage response kinases after release from mitotic arrest enhances the effects of microtubule poisons. (*a*) Experimental protocol. (*b*) Selective inhibition of PIKKs affects DNA damage signalling after a mitotic arrest. Samples were analysed by immunoblotting using the specified antibodies; asterisk denotes a non-specific signal on pS15-p53 blot. (*c,d*) Inhibition of PIKKs affects cell cycle progression following mitotic arrest. Cells arrested in mitosis for 2 h with nocodazole (*c*) or normal mitotic cells (*d*) were released as depicted in (*a*) and analysed using flow cytometry. The cumulative histograms show the percentage of cells in the different phases of the cell cycle, according to BrdU incorporation and DNA content. Data shown are from a representative experiment (*n* ≥ 3). (*e,f*) Inhibition of PIKKs reduces the viability and proliferation of cells following a mitotic arrest. (*e*) Cells treated as indicated in (*a*) were incubated with an FAM-DEVD-fmk probe to identify apoptotic cells and analysed by flow cytometry. The percentage of apoptotic cells is shown, values are means ± s.d. (*n* ≥ 3). Statistical differences were analysed with the Mann–Whitney test; n.s., non-significant, *p < 0.05, **p < 0.01 and ***p < 0.001. (*f*) The relative number of viable, adherent cells was determined by crystal violet assay. Values are means ± s.d. from quadruplicate wells of a representative experiment (*n* ≥ 3).

To address the significance of the DNA damage pathway activated in response to mitotic arrest, we analysed the effect of inhibiting ATM, ATR or DNA-PK after release from mitosis using the chemical inhibitors KU55933 [24], NU6027 [25] and NU7441 [26,27], respectively (for protocol, see figure 6*a*). Chemical inhibitors have an advantage over genetic approaches, because kinases can be targeted at specific points in the cell cycle; however, there remains the caveat that their cellular effects might be mediated by inhibition of other enzymes, especially other kinases (e.g. inhibition of CDK2 by NU6027 [25]). The relative selectivity of KU55933 and NU6027 was confirmed by their ability to block the phosphorylation of the ATM substrate Chk2 and the ATR substrate Chk1, respectively, as well as the selective inhibition of ATM autophosphorylation at S1981 by KU55933 but not NU6027 or NU7441 (figure 6*b*). We found that inhibition of ATM or DNA-PK in particular strongly suppressed the γH2AX signal generated immediately after release from mitosis, with a weaker effect by the ATR inhibitor. In addition, the ATM and ATR inhibitors both impaired p53 Ser15 phosphorylation and p21 induction at this time (figure 6*b*, left panels). We conclude that ATM, ATR and DNA-PK all contribute to DNA damage signalling after release from mitotic arrest.

Conversely, while the overall DDR had declined 24 h after release from mitotic arrest without kinase inhibitors, the γH2AX signal at this time was strongly enhanced by inhibition of ATM or ATR, and was maintained to a lesser extent by inhibition of DNA-PK. Each of the three inhibitors also induced further induction of p53 and p21 (figure 6*b*, right panels). This indicates that ATM, ATR and DNA-PK all play roles in recovery from the initial damage, which is prolonged when one or other is inhibited. Inhibiting each of these PIKKs reduced the ability of U2OS cells released from mitosis to enter S phase (figure 6*c,d*). This effect was more pronounced in cells that had been arrested in mitosis for 2 h (figure 6*c*) than in cells that had been synchronized in a normal mitosis (figure 6*d*). Furthermore, PIKK inhibitors increased the number of apoptotic cells in cultures that had been arrested in mitosis by nocodazole (figure 6*e*; electronic supplementary material, figure S7A,B) or taxol (electronic supplementary material, figure S7C,D) prior to release into interphase. Consistent with these effects, PIKK inhibitors restrained the proliferation of cells that had been arrested previously in mitosis (figure 6*f*; electronic supplementary material, figure S7B). Thus, ATM, ATR and DNA-PK each play pro-survival roles in interphase following mitotic stress, and their inhibition leads to enhanced cell cycle arrest, increased apoptosis and a reduction in overall cell proliferation.

### 4.7. BH3 mimetics and inhibitors of the DNA damage response act synergistically to sensitize cancer cells to microtubule poisons

**Figure 7.**
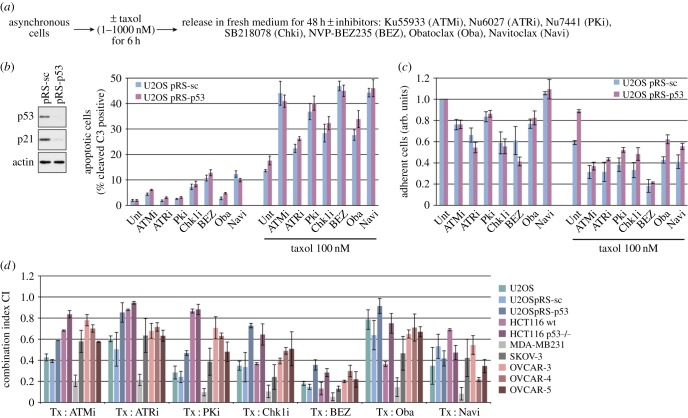
BH3 mimetics and inhibitors of the mitotic DNA damage response sensitize cancer cells synergistically to the effects of a microtubule poison. (*a*) Experimental protocol. (*b,c*) BH3 mimetics and inhibitors of the DNA damage response sensitize U2OS cells to taxol. U2OS cells in which p53 was stably depleted by shRNA (pRS-p53, confirmed by Western blot analysis) and control cells transfected with a scrambled shRNA (pRS-sc) were treated after release from taxol as indicated in (*a*). Apoptosis and proliferation of cells were followed in an IncuCyte. Values are means ± s.d. from quadruplicate wells of a representative experiment (*n* ≥ 3). (*d*) BH3 mimetics and inhibitors of the DNA damage response synergize with taxol. Indicated cell lines were treated as depicted in (*a*) first with taxol and then at least three different doses of the indicated inhibitor (see electronic supplementary material, experimental procedures) to determine their combination indexes (CIs) according to Chou [20]. The graphs show the CIs ± s.d. (*n* ≥ 3) obtained for 100 nM taxol treatments of U2OS and MDA-MB231 cells and 10 nM treatments of HCT116, SKOV-3 and OVCAR-3, 4, 5 cells. The CI values obtained at given concentrations depict an antagonism between the drugs when >1, an additive effect when equal to 1 and a synergism when <1.

We found that inhibitors of ATM, ATR and DNA-PK, including NVP-BEZ235, which also inhibits phosphatidyl-3-kinase and mTOR [28], as well as a Chk1 inhibitor (SB218078 [29]), all enhanced the induction of apoptosis in U2OS cells pre-treated for 6 h with taxol. So did Obatoclax or another BH3 mimetic Navitoclax (ABT-263) that selectively inhibits Bcl-2 and Bcl-x_L_ [30] (figure 7*a,b*). Conversely, taxol strongly enhanced the induction of apoptosis by both kinase inhibitors and BH3 mimetics because these drugs caused only low levels of apoptosis in the absence of taxol. Each of the kinase inhibitors and BH3 mimetics decreased the total number of U2OS cells whether or not the cells expressed p53, although suppression of p53 reduced the inhibition of cell proliferation by taxol (figure 7*c*).

We analysed the proliferative response of a variety of cancer cells from the NCI-60 cell lines panel using the Chou–Talalay method [20] to assess quantitatively the synergy between drugs (see electronic supplementary material, experimental procedures). Proliferation indexes corresponding to the combination indexes (CIs) plotted in figure 7*d* are shown in electronic supplementary material, figure S8. We found that there was a synergistic inhibitory effect (CI < 1) of taxol in combination with either BH3 mimetics or inhibitors of DDR kinases on the proliferation of many of the cell lines (CI curves obtained in U2OS cells are given in electronic supplementary material, figure S9), although there were significant differences between them (figure 7*d*; electronic supplementary material, table S2). Navitoclax had a very potent inhibitory effect on the proliferation of all the cell lines, but only when combined with taxol, indicating that this may be a generally effective combination therapy. Furthermore, NVP-BEZ235 produced a very strongly synergistic inhibitory effect with taxol in all of the cell lines, including those lacking p53.

## 5. Discussion

In this report, we have characterized a temporally controlled mitotic stress response pathway that is regulated by the oncogenic proteins Mcl-1 and Bcl-2/Bcl-x_L_. We show that this pathway determines the sensitivity of cancer cells to microtubule poisons and can be manipulated by combinations of these drugs with inhibitors of Bcl-2 family proteins or DDR kinases.

**Figure 8.**
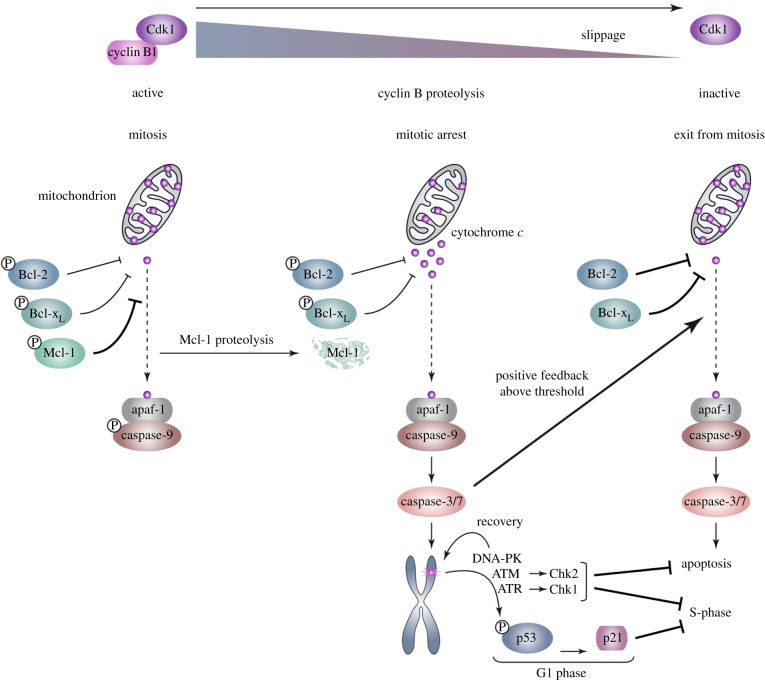
Cellular responses to microtubule poisons are determined by caspase-dependent DNA damage signalling initiated during delayed mitosis. During normal mitosis, release of cytochrome *c* from mitochondria is inhibited by the action of Bcl-2, Bcl-x_L_ and Mcl-1; downstream caspase activation is restrained by the inhibitory phosphorylation of caspase-9. Increasing phosphorylation of Bcl-2 and Bcl-x_L_, however, reduces their activity, whereas phosphorylation of Mcl-1 by CDK1–cyclin B initiates its proteolytic destruction. After a prolonged mitotic arrest, this results in the partial release of cytochrome *c* from mitochondria and the subapoptotic activation of caspase-3/7 when coupled with a slow decline in CDK1–cyclin B kinase activity and dephosphorylation of caspase-9. Caspase-3/7 activity results in localized DNA damage and activation of ATM, ATR and DNA-PK. The mitotic DNA damage response is amplified as cells slip out of mitosis, resulting in the phosphorylation of p53 and induction of p21, which inhibits CDK activity required for S-phase. ATM and ATR also activate Chk2 and Chk1, respectively, which inhibit subsequent cell cycle progression. ATM, ATR and DNA-PK also have functions in recovery from telomere damage and restrain apoptosis.

Regulation of the mitotic stress response by Bcl-2 family proteins suggests a mechanism for temporal control of the initiation of the response (figure 8). Cells in mitosis are restricted in their ability to induce new transcription and changes during mitosis are mediated primarily through post-translational mechanisms, notably protein phosphorylation and protein degradation by the ubiquitin–proteasome system. Mcl-1 is destroyed after a prolonged mitotic arrest [11], and loss of Mcl-1 is likely to be a key step in the initiation of the response. Although Bcl-2 and Bcl-x_L_ are stable during mitotic arrest, they are highly phosphorylated, and this phosphorylation has been associated with the inhibition of their function [13]. Nevertheless, cells arrested in mitosis are exquisitely sensitive to Navitoclax [31] (D.J.C., K.O.H., L.A.A. & P.R.C. 2015, unpublished data), showing that Bcl-2 and/or Bcl-x_L_ are critical to prevent the induction of mitotic stress when Mcl-1 has been degraded. It seems, therefore, that the threshold to induce caspase activation is reduced in mitotically arrested cells through both the destruction of Mcl-1 and the partial inhibition of Bcl-2/Bcl-x_L_.

Subapoptotic activation of caspase-3/7 is likely to require cytochrome c release from mitochondria, because it is controlled by Bcl-2 family proteins that function at this step of the pathway; it is unlikely, however, that there is widespread loss of mitochondrial outer membrane integrity, because the cells mostly remain viable. The ability of such cells to survive a low-level activation of caspase-3/7 also indicates that there are mechanisms to prevent conversion to full apoptosis, possibly through suppression of auto-amplification mechanisms that otherwise produce full caspase-3/7 activation, such as the inhibitory phosphorylation of caspase-9 [2] and caspase-2 [32] during mitosis. When mitotically delayed cells exit mitosis, a higher threshold for full apoptosis is likely to be restored as Bcl-2 and Bcl-x_L_ are dephosphorylated and Mcl-1 levels recover through new synthesis. We propose that recovery mechanisms also reduce caspase-3/7 activity to non-stressed levels during interphase, perhaps through the activity of inhibitory proteins (IAPs) and/or the proteolytic turnover of the activated caspases. The majority of mitotically stressed cells are likely to survive with most of their constituent mitochondria intact, as has been observed in response to other apoptotic stimuli [33].

Subapoptotic caspase activity might induce a DDR in mitotically arrested cells through the generation of DNA strand breaks in a restricted manner by the apoptotic endonuclease CAD after cleavage of its inhibitor ICAD [17,34]. Interestingly, recent work has shown that a DDR is initiated at telomeres during a prolonged mitotic arrest [15], and telomeres might be particularly sensitive to CAD-dependent DNA strand breaks; alternatively, caspase-dependent cleavage of other proteins might lead to telomere deprotection. The repair of DNA double-stranded breaks is inhibited during mitosis, which prevents telomere fusions [35] and may allow CAD-generated breaks to accumulate during mitotic arrest. Subsequent signalling is likely to involve the recruitment of secondary factors to sites marked by γH2AX when cells exit mitotic arrest [35,36].

In addition to the DDR induced at specific foci during mitosis, massive DNA damage induced on individual lagging chromosomes during release from mitosis (an effect that, by contrast to foci formation, does not appear to be dependent on the period of prior mitotic arrest [37]) is likely to contribute to the effect of mitotic disruption in certain individual cells [37,38]. Furthermore, failure to complete nuclear envelope assembly in telophase is coupled to a widespread DDR in micronuclei [39]. Nevertheless, caspase-dependent DNA damage, which is restrained by Mcl-1 together with Bcl-2/Bcl-x_L_ during normal mitosis, appears to be a major mechanism by which a prolonged mitotic delay affects subsequent cell cycle progression and cell proliferation.

The role of p53 in cellular responses to microtubule poisons has previously been unclear, but from our results it is now apparent that phosphorylation of the protein on Ser15, which is associated with the activation of its transcriptional activity [40], is induced by these drugs because of mitotic stress. We have found that, although Ser15 phosphorylation and the p53-dependent induction of p21 in response to microtubule poisons are blocked by inhibitors of the PIKKs, the induction of p53 protein is not, indicating a distinct mechanism for its transcriptional induction and/or stabilization of the p53 protein as a consequence of mitotic arrest. Downstream of p53, it is very likely that, in addition to p21, effectors involved in the control of cell proliferation or survival are induced transcriptionally in response to mitotic stress. Loss of p53 function during tumourigenesis might permit continued cancer cell proliferation by removing the G1 checkpoint following mitotic stress. Although we find that the increase in proliferation rate following depletion of p53 in U2OS cells (which are a useful model for p53 responses) is counteracted by an increase in apoptosis, in other cell types, this balance might differ owing to additional genetic changes, such that loss of p53 function could result in increased cell survival after the disruption of mitosis and might alter sensitivity to anti-mitotic drugs.

We propose that the caspase-dependent mitotic DNA damage pathway plays a role in the maintenance of normal chromosome fidelity and suppression of aneuploidy by inhibiting the proliferation and survival of aberrant cells. Conversely, suppression of this mitotic stress response in cancer cells is likely to sustain them by permitting continued proliferation despite delayed mitoses owing to polyploidy, aneuploidy or supernumerary centrosomes [41]. Inhibition of the mitotic stress response by Mcl-1 and Bcl-2/Bcl-x_L_ suggests that these proteins can promote oncogenesis when they are over-expressed or stabilized not only through the prevention of apoptosis, but also through the suppression of both mitotic DNA damage signalling and subsequent activation of p53. Ordinarily, this response is likely to induce cell senescence as a consequence of a sustained G1 arrest, although not in cancer cells that are resistant to senescence, for instance, owing to loss of p14*^ARF^*(as in the U2OS cells used in our experiments [42]). Thus, inhibition of the mitotic stress response and the suppression of apoptosis might together permit the proliferation of cells with the increased rates of chromosome abnormalities commonly found in cancer.

Our results have potential implications for anti-cancer therapy. A very prolonged arrest in mitosis induced by a high concentration of a microtubule poison or ablation of Cdc20 can lead directly to cell death in some cell types [6,8,9], but such a prolonged arrest is unlikely to be sustained in patients undergoing chemotherapy, in which case the response of cancer cells to suboptimal doses of the drug and a delay (as opposed to an arrest) in mitosis are likely to be important for clinical effectiveness. We show that the mitotic stress response can be hyperactivated by a BH3 mimetic, which promotes the DDR, delays subsequent cell cycle progression and enhances apoptosis, thereby inhibiting cancer cell proliferation. The combination of a BH3 mimetic with taxol might therefore achieve an improved therapeutic index against proliferating cancer cells, particularly those in which the response to mitotic stress is suppressed by overexpression of Mcl-1 or another inhibitor of caspase activation.

We also find that inhibition of DDR kinases after mitotic stress has a similar overall effect to the BH3 mimetic, suggesting that the kinase inhibitors act by preventing recovery mechanisms resulting in a prolonged or enhanced response. This is evidenced by the strong DNA damage signals that accumulate after release from mitotic arrest when individual PIKKs are inhibited. Remarkably, there seems to be a commonality of cellular responses between apparently non-genotoxic anti-cancer drugs that target microtubules and genotoxic drugs or radiation that directly damage DNA because the former also induce a DDR through mitotic stress. Thus, drugs that target components of the DDR or DNA repair could enhance the effectiveness of anti-mitotic drugs.

## Supplementary Material

Supplementary Tables and Figures

## References

[1] Foley EA Kapoor TM 2013 Microtubule attachment and spindle assembly checkpoint signalling at the kinetochore. Nat. Rev. Mol. Cell Biol. 14, 25–37 (doi:10.1038/nrm3494) 23258294 10.1038/nrm3494 PMC3762224

[2] Allan LA Clarke PR 2007 Phosphorylation of caspase-9 by CDK1/cyclin B1 protects mitotic cells against apoptosis. Mol. Cell 26, 301–310 (doi:10.1016/j.molcel.2007.03.019) 17466630 10.1016/j.molcel.2007.03.019

[3] Bekier ME Fischbach R Lee J Taylor WR 2009 Length of mitotic arrest induced by microtubule-stabilizing drugs determines cell death after mitotic exit. Mol. Cancer Ther. 8, 1646–1654 (doi:10.1158/1535-7163.MCT-08-1084) 19509263 10.1158/1535-7163.MCT-08-1084 PMC12337704

[4] Huang HC Shi J Orth JD Mitchison TJ 2009 Evidence that mitotic exit is a better cancer therapeutic target than spindle assembly. Cancer Cell 16, 347–358 (doi:10.1016/j.ccr.2009.08.020) 19800579 10.1016/j.ccr.2009.08.020 PMC2758291

[5] Brito DA Rieder CL 2006 Mitotic checkpoint slippage in humans occurs via cyclin B destruction in the presence of an active checkpoint. Curr. Biol. 16, 1194–1200 (doi:10.1016/j.cub.2006.04.043) 16782009 10.1016/j.cub.2006.04.043 PMC2749311

[6] Gascoigne KE Taylor SS 2008 Cancer cells display profound intra- and interline variation following prolonged exposure to antimitotic drugs. Cancer Cell 14, 111–122 (doi:10.1016/j.ccr.2008.07.002) 18656424 10.1016/j.ccr.2008.07.002

[7] Rieder CL Maiato H 2004 Stuck in division or passing through: what happens when cells cannot satisfy the spindle assembly checkpoint. Dev. Cell 7, 637–651 (doi:10.1016/j.devcel.2004.09.002) 15525526 10.1016/j.devcel.2004.09.002

[8] Shi J Orth JD Mitchison T 2008 Cell type variation in responses to antimitotic drugs that target microtubules and kinesin-5. Cancer Res. 68, 3269–3276 (doi:10.1158/0008-5472.CAN-07-6699) 18451153 10.1158/0008-5472.CAN-07-6699

[9] Manchado E 2010 Targeting mitotic exit leads to tumor regression *in vivo*: modulation by Cdk1, Mastl, and the PP2A/B55α,δ phosphatase. Cancer Cell 18, 641–654 (doi:10.1016/j.ccr.2010.10.028) 21156286 10.1016/j.ccr.2010.10.028

[10] Clarke PR Allan LA 2009 Cell-cycle control in the face of damage: a matter of life or death. Trends Cell Biol. 19, 89–98 (doi:10.1016/j.tcb.2008.12.003) 19168356 10.1016/j.tcb.2008.12.003

[11] Harley ME Allan LA Sanderson HS Clarke PR 2010 Phosphorylation of Mcl-1 by CDK1–cyclin B1 initiates its Cdc20-dependent destruction during mitotic arrest. EMBO J. 29, 2407–2420 (doi:10.1038/emboj.2010.112) 20526282 10.1038/emboj.2010.112 PMC2910263

[12] Wertz IE 2011 Sensitivity to antitubulin chemotherapeutics is regulated by MCL1 and FBW7. Nature 471, 110–114 (doi:10.1038/nature09779) 21368834 10.1038/nature09779

[13] Terrano DT Upreti M Chambers TC 2010 Cyclin-dependent kinase 1-mediated Bcl-xL/Bcl-2 phosphorylation acts as a functional link coupling mitotic arrest and apoptosis. Mol. Cell. Biol. 30, 640–656 (doi:10.1128/MCB.00882-09) 19917720 10.1128/MCB.00882-09 PMC2812246

[14] Uetake Y Sluder G 2010 Prolonged prometaphase blocks daughter cell proliferation despite normal completion of mitosis. Curr. Biol. 20, 1666–1671 (doi:10.1016/j.cub.2010.08.018) 20832310 10.1016/j.cub.2010.08.018 PMC2946429

[15] Hayashi MT Cesare AJ Fitzpatrick JA Lazzerini-Denchi E Karlseder J 2012 A telomere-dependent DNA damage checkpoint induced by prolonged mitotic arrest. Nat. Struct. Mol. Biol. 19, 387–394 (doi:10.1038/nsmb.2245) 22407014 10.1038/nsmb.2245 PMC3319806

[16] Imreh G Norberg HV Imreh S Zhivotovsky B 2011 Chromosomal breaks during mitotic catastrophe trigger γH2AX-ATM-p53-mediated apoptosis. J. Cell Sci. 124, 2951–2963 (doi:10.1242/jcs.081612) 21878502 10.1242/jcs.081612

[17] Orth JD Loewer A Lahav G Mitchison TJ 2012 Prolonged mitotic arrest triggers partial activation of apoptosis, resulting in DNA damage and p53 induction. Mol. Biol. Cell 23, 567–576 (doi:10.1091/mbc.E11-09-0781) 22171325 10.1091/mbc.E11-09-0781 PMC3279386

[18] Crighton D O'Prey J Bell HS Ryan KM 2007 p73 regulates DRAM-independent autophagy that does not contribute to programmed cell death. Cell Death Differ. 14, 1071–1079 (doi:10.1038/sj.cdd.4402108) 17304243 10.1038/sj.cdd.4402108

[19] Aylon Y Michael D Shmueli A Yabuta N Nojima H Oren M 2006 A positive feedback loop between the p53 and Lats2 tumor suppressors prevents tetraploidization. Genes Dev. 20, 2687–2700 (doi:10.1101/gad.1447006) 17015431 10.1101/gad.1447006 PMC1578695

[20] Chou TC 2010 Drug combination studies and their synergy quantification using the Chou–Talalay method. Cancer Res. 70, 440–446 (doi:10.1158/0008-5472.CAN-09-1947) 20068163 10.1158/0008-5472.CAN-09-1947

[21] Jackson SP Bartek J 2009 The DNA-damage response in human biology and disease. Nature 461, 1071–1078 (doi:10.1038/nature08467) 19847258 10.1038/nature08467 PMC2906700

[22] So S Davis AJ Chen DJ 2009 Autophosphorylation at serine 1981 stabilizes ATM at DNA damage sites. J. Cell Biol. 187, 977–990 (doi:10.1083/jcb.200906064) 20026654 10.1083/jcb.200906064 PMC2806275

[23] Nguyen M 2007 Small molecule obatoclax (GX15-070) antagonizes MCL-1 and overcomes MCL-1-mediated resistance to apoptosis. Proc. Natl Acad. Sci. USA 104, 19 512–19 517 (doi:10.1073/pnas.0709443104) 10.1073/pnas.0709443104 PMC2148320 18040043

[24] Hickson I 2004 Identification and characterization of a novel and specific inhibitor of the ataxia-telangiectasia mutated kinase ATM. Cancer Res. 64, 9152–9159 (doi:10.1158/0008-5472.CAN-04-2727) 15604286 10.1158/0008-5472.CAN-04-2727

[25] Peasland A 2011 Identification and evaluation of a potent novel ATR inhibitor, NU6027, in breast and ovarian cancer cell lines. Br. J. Cancer 105, 372–381 (doi:10.1038/bjc.2011.243) 21730979 10.1038/bjc.2011.243 PMC3172902

[26] Leahy JJ Golding BT Griffin RJ Hardcastle IR Richardson C Rigoreau L Smith GC 2004 Identification of a highly potent and selective DNA-dependent protein kinase (DNA-PK) inhibitor (NU7441) by screening of chromenone libraries. Bioorg. Med. Chem. Lett. 14, 6083–6087 (doi:10.1016/j.bmcl.2004.09.060) 15546735 10.1016/j.bmcl.2004.09.060

[27] Zhao Y 2006 Preclinical evaluation of a potent novel DNA-dependent protein kinase inhibitor NU7441. Cancer Res. 66, 5354–5362 (doi:10.1158/0008-5472.CAN-05-4275) 16707462 10.1158/0008-5472.CAN-05-4275

[28] Toledo LI 2011 A cell-based screen identifies ATR inhibitors with synthetic lethal properties for cancer-associated mutations. Nat. Struct. Mol. Biol. 18, 721–727 (doi:10.1038/nsmb.2076) 21552262 10.1038/nsmb.2076 PMC4869831

[29] Jackson JR Gilmartin A Imburgia C Winkler JD Marshall LA Roshak A 2000 An indolocarbazole inhibitor of human checkpoint kinase (Chk1) abrogates cell cycle arrest caused by DNA damage. Cancer Res. 60, 566–572. 10676638

[30] Tse C 2008 ABT-263: a potent and orally bioavailable Bcl-2 family inhibitor. Cancer Res. 68, 3421–3428 (doi:10.1158/0008-5472.CAN-07-5836) 18451170 10.1158/0008-5472.CAN-07-5836

[31] Shi J Zhou Y Huang HC Mitchison TJ 2011 Navitoclax (ABT-263) accelerates apoptosis during drug-induced mitotic arrest by antagonizing Bcl-xL. Cancer Res. 71, 4518–4526 (doi:10.1158/0008-5472.CAN-10-4336) 21546570 10.1158/0008-5472.CAN-10-4336 PMC3129452

[32] Andersen JL 2009 Restraint of apoptosis during mitosis through interdomain phosphorylation of caspase-2. EMBO J. 28, 3216–3227 (doi:10.1038/emboj.2009.253) 19730412 10.1038/emboj.2009.253 PMC2771089

[33] Tait SW Parsons MJ Llambi F Bouchier-Hayes L Connell S Munoz-Pinedo C Green DR 2010 Resistance to caspase-independent cell death requires persistence of intact mitochondria. Dev. Cell 18, 802–813 (doi:10.1016/j.devcel.2010.03.014) 20493813 10.1016/j.devcel.2010.03.014 PMC3004027

[34] Enari M Sakahira H Yokoyama H Okawa K Iwamatsu A Nagata S 1998 A caspase-activated DNase that degrades DNA during apoptosis, and its inhibitor ICAD. Nature 391, 43–50 (doi:10.1038/34112) 9422506 10.1038/34112

[35] Orthwein A Fradet-Turcotte A Noordermeer SM Canny MD Brun CM Strecker J Escribano-Diaz C Durocher D 2014 Mitosis inhibits DNA double-strand break repair to guard against telomere fusions. Science 344, 189–193 (doi:10.1126/science.1248024) 24652939 10.1126/science.1248024

[36] Giunta S Belotserkovskaya R Jackson SP 2010 DNA damage signaling in response to double-strand breaks during mitosis. J. Cell Biol. 190, 197–207 (doi:10.1083/jcb.200911156) 20660628 10.1083/jcb.200911156 PMC2930281

[37] Janssen A van der Burg M Szuhai K Kops GJ Medema RH 2011 Chromosome segregation errors as a cause of DNA damage and structural chromosome aberrations. Science 333, 1895–1898 (doi:10.1126/science.1210214) 21960636 10.1126/science.1210214

[38] Crasta K 2012 DNA breaks and chromosome pulverization from errors in mitosis. Nature 482, 53–58 (doi:10.1038/nature10802) 22258507 10.1038/nature10802 PMC3271137

[39] Hatch EM Fischer AH Deerinck TJ Hetzer MW 2013 Catastrophic nuclear envelope collapse in cancer cell micronuclei. Cell 154, 47–60 (doi:10.1016/j.cell.2013.06.007) 23827674 10.1016/j.cell.2013.06.007 PMC3749778

[40] Loughery J Cox M Smith LM Meek DW 2014 Critical role for p53-serine 15 phosphorylation in stimulating transactivation at p53-responsive promoters. Nucleic Acid Res. 42, 7666–7680 (doi:10.1093/nar/gku501) 24928858 10.1093/nar/gku501 PMC4081099

[41] Yang Z Loncarek J Khodjakov A Rieder CL 2008 Extra centrosomes and/or chromosomes prolong mitosis in human cells. Nat. Cell Biol. 10, 748–751 (doi:10.1038/ncb1738) 18469805 10.1038/ncb1738 PMC2430725

[42] Park YB Park MJ Kimura K Shimizu K Lee SH Yokota J 2002 Alterations in the *INK4a/ARF* locus and their effects on the growth of human osteosarcoma cell lines. Cancer Genet. Cytogenet. 133, 105–111 (doi:10.1016/S0165-4608(01)00575-1) 11943335 10.1016/s0165-4608(01)00575-1

