# Supplementary material for: Cellular responses to a prolonged delay in mitosis are determined by a DNA damage response controlled by Bcl-2 family proteins
Source: Open Biol. 2015 Mar 11;5(3):140156. doi: 10.1098/rsob.140156 (PMC4389791; doi:10.1098/rsob.140156)
Supplement: Supplementary Tables and Figures [file rsob140156supp1.pdf]

SUPPLEMENTAL INFORMATION

Additional Experimental Procedures

Cell culture

Cells were maintained in Dulbecco's modified Eagle's medium or RPMI (OVCAR-3, 4 and 5 cells) (Invitrogen) containing 10 % foetal bovine serum (Biosera), 50 IU/mL Penicillin and 50 µg/mL streptomycin (Invitrogen). Cells were grown at 37°C with 5 % CO_2, periodically tested to be free from mycoplasma and their passage number did not exceed 20.

Cell synchronisation by double thymidine block

U2OS cells were treated with 2 mM thymidine for 16 h, released for 8 h in fresh media before adding 2 mM thymidine for a further 16 h. Cells synchronised at the G1/S boundary were then released in fresh media.

Western blot analyses

Sample were prepared in a RIPA buffer (50 mM Tris, 150 mM NaCl, 0.1 % SDS, 0.5 % Sodium deoxycholate, 1 % Igepal CA630, 2 mM EDTA, 50 mM NaF, pH 8) and titrated using the DC Protein Assay (Biorad). 5 to 20 µg were separated by SDS-PAGE and transferred onto nitrocellulose membranes (Amersham). Membranes were blocked 1 h at room temperature, incubated overnight at 4°C with primary antibodies according to Supplemental Table 1, incubated for 1 h with secondary antibodies and developed using standard ECL protocol.

Flow cytometry

Cells were incubated with 30 µM BrdU for 15 min and fixed in 80 % ethanol overnight at -20°C. Pelleted cells were incubated in 0.5 mg/mL pepsin in 30 mM HCl for 1 h at 37°C, centrifuged and incubated for 15 min in 2 M HCl. Cells were then washed twice in a blocking buffer (0.5 % BSA in PBS) and incubated with a mouse anti-BrdU (1/60 in 0.5 % Tween 20 in blocking buffer, BD Biosciences 555627) for 1 h at room temperature. After a wash, BrdU incorporation was detected with a FITC-conjugated anti-mouse secondary antibody (1/100 in

blocking buffer, Sigma-Aldrich) for 1 h at room temperature. Cells were finally counterstained for DNA content with a solution containing 50 µg/mL propidium iodide and 200 µg/mL RNAse A in PBS for 30 min at 37°C. Samples were analysed by flow cytometry (FACScan, BD Biosciences) and Flowjo (Tree Star).

For pSer10-Histone H3 immuno-staining, cells were fixed as described above, washed in a blocking buffer (0.5 % BSA in PBS) and blocked for a further 10 min at room temperature. Cells were then incubated in a mouse anti-pSer10-Histone H3 (1/150 in blocking buffer, 05-806, Millipore), washed twice and resuspended in a FITC-conjugated anti-mouse secondary antibody (1/25 in blocking buffer, Dako) for 1 h at room temperature. After propidium iodide staining, samples were analysed by flow cytometry as described above.

To determine the number of apoptotic cells in a population, a Fluorochrome Labeled Inhibitor of caspases (FLICA) staining was used according to the manufacturer's instructions (Immunochemistry Technologies). Briefly, adherent and floating cells were collected after indicated times and treatments, incubated with the caspase-3/7 substrate FAM-DEVD-fmk in PBS for 1 h at 37°C under 5 % CO_2, washed twice in the wash buffer provided and analysed by flow cytometry. Data were analysed with Flowjo and the percentages of highly fluorescent apoptotic cells were determined on Side Scatter / FL1 (FITC channel) dot plots.

Proliferation assays

Cells seeded in 96-well plates were monitored over time by high definition phase contrast in an Incucyte ZOOM (Essen). Cell growth was estimated using the built-in software that provides a rate of cell confluence based on segmentation of label-free phase contrast images. Apoptosis was assessed by treating cells with NucView 488 (Biotium) at the beginning of the time course. According to the manufacturer's instructions, the amount of fluorescent apoptotic cells was measured after 48 h of treatment and expressed as percentages of DNA-containing objects revealed by a Vybrant DyeCycle Green DNA staining (Invitrogen).

Synergies between drugs were determined according to the Chou-Talalay method [20] using a crystal violet based assay. We distinguished 2 sets of cells in regard to their sensitivity towards taxol, resistant U2OS and MDA-MB231 cells in one hand and sensitive HCT116, SKOV-3 and OVCAR-3, 4, 5 cells. These set of cells were respectively treated with concentrations of taxol ranging from 10 to 1000 nM and from 1 to 100 nM to surround the half maximal effective concentration (EC50) of taxol (Supp Table 1) but the concentrations of the different inhibitors studied were the same. Thus, the doses used for the ATMi and ATRi ranged from 1 to 100 µM, the DNA-PKi (PKi) and Chk1i from 0.1 to 10 µM, the pan-PIKKs

inhibitor BEZ and Obatoclax from 5 to 500 nM, and Navitoclax from 20 to 2000 nM. The combinations were also kept at constant ratios allowing automated computer simulation of the combination Index (CI) as shown in Supp. Fig. 9. The software CalcuSyn (Biosoft) was used to analyse the data on at least three doses per assay, to calculate the EC50s, and the CIs. The CI values obtained at given concentrations depict an antagonism between the drugs when >1, an additive effect when equal to 1 and a synergism when <1.

Immunofluorescence assays and microscopy

U2OS cells collected as indicated were fixed in 2 % paraformaldehyde in PBS for 15 min at room temperature, washed in PBS and stored at 4°C. Cells were then cytospun in a Cytospin 3 (Thermo Scientific) at 1000 rpm for 4 min (20000 cells per spot), permeabilised in a KCM buffer (10 mM Tris pH 7.5, 120 mM KCl, 20 mM NaCl, 0.1 % (v/v) Triton X100) for 10 min at room temperature and blocked in an antibody dilution buffer (20 mM Tris pH 7.5, 150 mM NaCl, 2 % (w/v) BSA, 2 % (w/v) nonfat dry milk, 0.2 % (v/v) fish gelatin, 0.1 % Triton X100) for 20 min at 37°C. Slides were incubated in a rabbit anti-γH2AX antibody (1/1000, sc101696, Santa Cruz) in blocking buffer overnight at 4°C. After 3 washes in 0.1 % Tween 20 in PBS, slides were incubated in an Alexa 488-conjugated anti-rabbit secondary antibody (1/500, Molecular Probes) for 1 h at room temperature, washed 3 times and counterstained with DAPI. Cells were imaged using a Leica DM5000B through a 40X objective. γH2AX foci were counted using ImageJ (NIH) and the macro PZ-FociEZ (www.pzfociez.com) in at least 200 cells per treatment, and their distributions were fitted with a Gaussian regression using GraphPad Prism.

Supplemental Table 1

Primary antibodies used for Western blotting in this study.

Epitope	Manufacturer	Reference	Proteins (μg)	Blocking	Dilution	Diluant
β-Actin	Sigma-Aldrich	A2066	2	5% Milk	1/2000	5% Milk
ATM	Cell Signalling	#2873	5	5% BSA	1/1000	5% BSA
pS1981-ATM	Abcam	ab81292	5	5% Milk	1/1000	5% Milk
Chk1	Santa-Cruz	sc-8408	5	5% Milk	1/1000	5% Milk
pS345-Chk1	Cell Signalling	#2348	5	5% Milk	1/1000	5% BSA
Chk2	Millipore	25-649	5	5% Milk	1/1000	5% Milk
pT68-Chk2	Abcam	ab38461	15	5% Milk	1/500	5% Milk
Cyclin B1	Santa-Cruz	sc-752	2	5% Milk	1/1000	5% Milk
pS139-(γ)H2AX	Santa-Cruz	sc-101696	5	5% BSA	1/500	5% BSA
pS10-H3	Millipore	06-570	2	5% Milk	1/2000	5% Milk
Mcl-1	BD Biosciences	559027	5	5% Milk	1/500	5% Milk
p21	Santa-Cruz	sc-756	5	5% Milk	1/500	2.5% BSA/2.5% Milk
p53	Abcam	ab1101	5	5% Milk	1/1000	5% Milk
pS15-p53	Cell Signalling	#9284	5	5% Milk	1/1000	5% BSA
PARP	AbD Serotec	MCA1522G	5	5% Milk	1/1000	5% Milk

Additional Results

Supplemental Table 2

Half maximal effective concentrations (EC50) of taxol, BH3 mimetics and inhibitors of the DNA damage response towards the cancer cell lines used in this study.

EC50s of taxol were determined by treating asynchronous cultures of indicated cell lines with six concentrations of taxol (Tx, 1-1000 nM) for 6 h then released in fresh media for 48 h. EC50s of the other drugs were determined by treating cells for 48 h with six concentrations of DNA damage signalling kinase inhibitors ATMi (1-100 µM), ATRi (1-100 µM), DNA-PKi (PKi, 0.1-10 µM), Chk1i (0.1-10 µM), BEZ (5-500 nM) or BH3 mimetics Obatoclax (Oba, 5-500 nM) or Navitoclax (Navi, 20- 2000 nM). The relative numbers of live adherent cells were quantified by crystal violet assay and the software Calcusyn was used to generate dose-effect curves and to determine EC50. Values are means ± SD, n≥3.

EC50 ± SD	Tx (nM)	ATMi (µM)	ATRi (µM)	PKi (µM)	Chk1i (µM)	BEZ (nM)	Oba (nM)	Navi (µM)
U2OS	272.4 ± 30.6	18.9 ± 5.9	20.7 ± 10.8	6.7 ± 0.8	3.6 ± 1.3	56.9 ± 5.8	81.1 ± 5.5	1.1 ± 0.1
U2OS pRS-sc	291.8 ± 48.7	19.8 ± 3.4	16.3 ± 8.3	7.6 ± 2.4	3.1 ± 0.2	66.6 ± 12.5	100.4 ± 14.0	1.3 ± 0.2
U2OS pRS-p53	479.2 ± 61.5	17.5 ± 1.3	11.9 ± 2.2	4.5 ± 0.6	2.1 ± 0.9	32.0 ± 7.9	111.1 ± 12.9	1.3 ± 0.2
HCT116 wt	10.9 ± 0.4	38.1 ± 4.9	48.2 ± 1.6	4.9 ± 0.1	4.1 ± 0.1	52.3 ± 12.6	54.0 ± 19.3	1.6 ± 0.1
HCT116 p53 -/-	21.0 ± 2.1	20.6 ± 3.8	36.8 ± 3.6	3.3 ± 0.5	7.7 ± 4.8	33.2 ± 1.9	69.1 ± 18.2	1.4 ± 0.1
MDA-MB231	183.3 ± 20.3	31.2 ± 2.8	35.8 ± 5.4	8.5 ± 1.1	3.8 ± 0.8	73.0 ± 24.7	212.3 ± 9.9	1.5 ± 0.1
SKOV-3	10.6 ± 3.1	38.0 ± 10.0	28.4 ± 4.5	5.9 ± 0.4	10.7 ± 6.2	59.9 ± 12.2	208.3 ± 62.8	1.3 ± 0.1
OVCAR-3	28.0 ± 4.7	28.9 ± 2.4	49.2 ± 6.9	8.7 ± 2.2	7.9 ± 0.1	18.3 ± 11.0	226.8 ± 38.0	1.5 ± 0.1
OVCAR-4	23.7 ± 3.7	30.4 ± 3.8	17.4 ± 3.7	7.8 ± 1.5	7.8 ± 0.2	90.9 ± 8.8	150.3 ± 19.0	1.8 ± 0.1
OVCAR-5	46.6 ± 8.0	34.6 ± 8.2	41.0 ± 2	9.7 ± 2.3	5.0 ± 0.1	54.4 ± 14.2	154.5 ± 71.9	2.1 ± 0.2

Figure S1

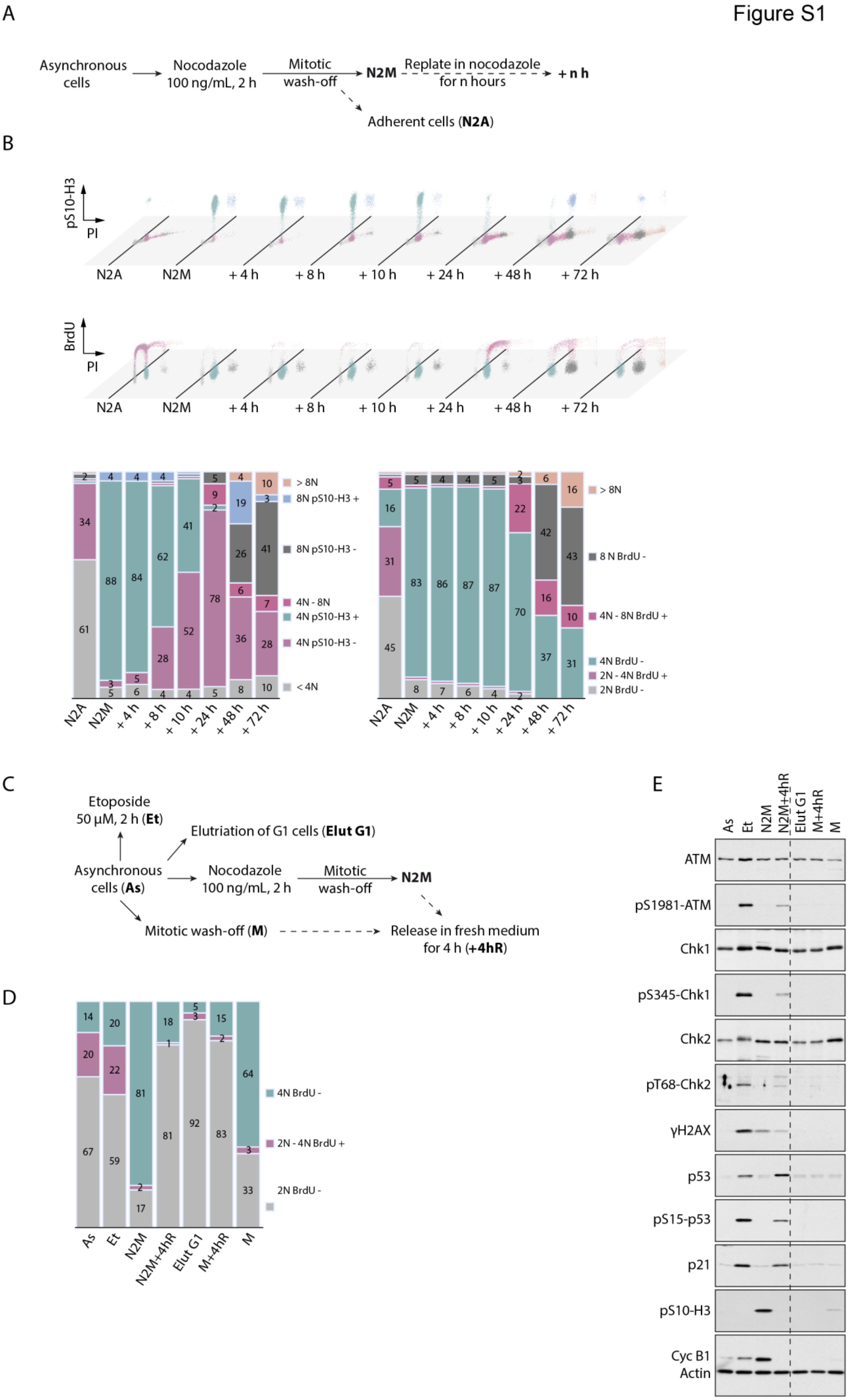

A
Asynchronous cells
Nocodazole 100 ng/mL, 2 h
Mitotic wash-off
N2M
Replate in nocodazole for n hours
+ n h
Adherent cells (N2A)
B
pS10-H3
PI
BrdU
N2A
N2M
+ 4 h
+ 8 h
+ 10 h
+ 24 h
+ 48 h
+ 72 h
> 8N
8N pS10-H3 +
8N pS10-H3 -
4N - 8N
4N pS10-H3 +
4N pS10-H3 -
< 4N
8 N BrdU -
4N - 8N BrdU +
4N BrdU -
2N - 4N BrdU +
2N BrdU -
C
Etoposide 50 µM, 2 h (Et)
Elutriation of G1 cells (Elut G1)
Asynchronous cells (As)
Nocodazole 100 ng/mL, 2 h
Mitotic wash-off
N2M
Mitotic wash-off (M)
Release in fresh medium for 4 h (+4hR)
D
4N BrdU -
2N - 4N BrdU +
2N BrdU -
As
Et
N2M
N2M+4hR
Elut G1
M+4hR
M
E
ATM
pS1981-ATM
Chk1
pS345-Chk1
Chk2
pT68-Chk2
γH2AX
p53
pS15-p53
p21
pS10-H3
Cyc B1
Actin

Figure S1. Mitotic delay induces a DNA damage response in synchronised cells.

A. Experimental protocol. Following a pre-synchronisation for 2 h in nocodazole (100 ng/mL), mitotic U2OS cells were collected (N2M) and either replated in nocodazole or released in fresh media for indicated times. After wash-off, remaining adherent cells were collected to control the interphase effects of nocodazole (N2A).

B. Fate of U2OS cells during a prolonged mitotic arrest. Cells arrested in mitosis and slipped cells as described in **A** were analysed by immunostaining followed by flow cytometry analyses. Cell cycle profiles were determined using pSer10-Histone H3 staining (mitotic marker) and BrdU staining (S phase marker) followed by a propidium iodide (PI) co-staining (DNA content). On top, FACS profiles are plotted showing colour-coded cell populations; below, cumulative histograms show the percentages of cells in different phases of the cell cycle related to the FACS profiles shown above. Data shown are from a representative experiment repeated three times.

C. Protocol for analysis of the effects of nocodazole on interphase cells. G1 cells obtained after releasing for 4 h (+4hR), washed-off mitotic cells (M) from asynchronous untreated cultures or from mitotically-arrested cells with nocodazole for 2 h (N2M) were compared to G1 cells isolated by centrifugal elutriation (Elut G1).

D, E. Synchronised interphase samples were collected as indicated in **C** and cell cycle progression was followed by propidium iodide staining and flow cytometry analyses **(D)** or analysed by immunoblotting using the specified antibodies **(E)**.

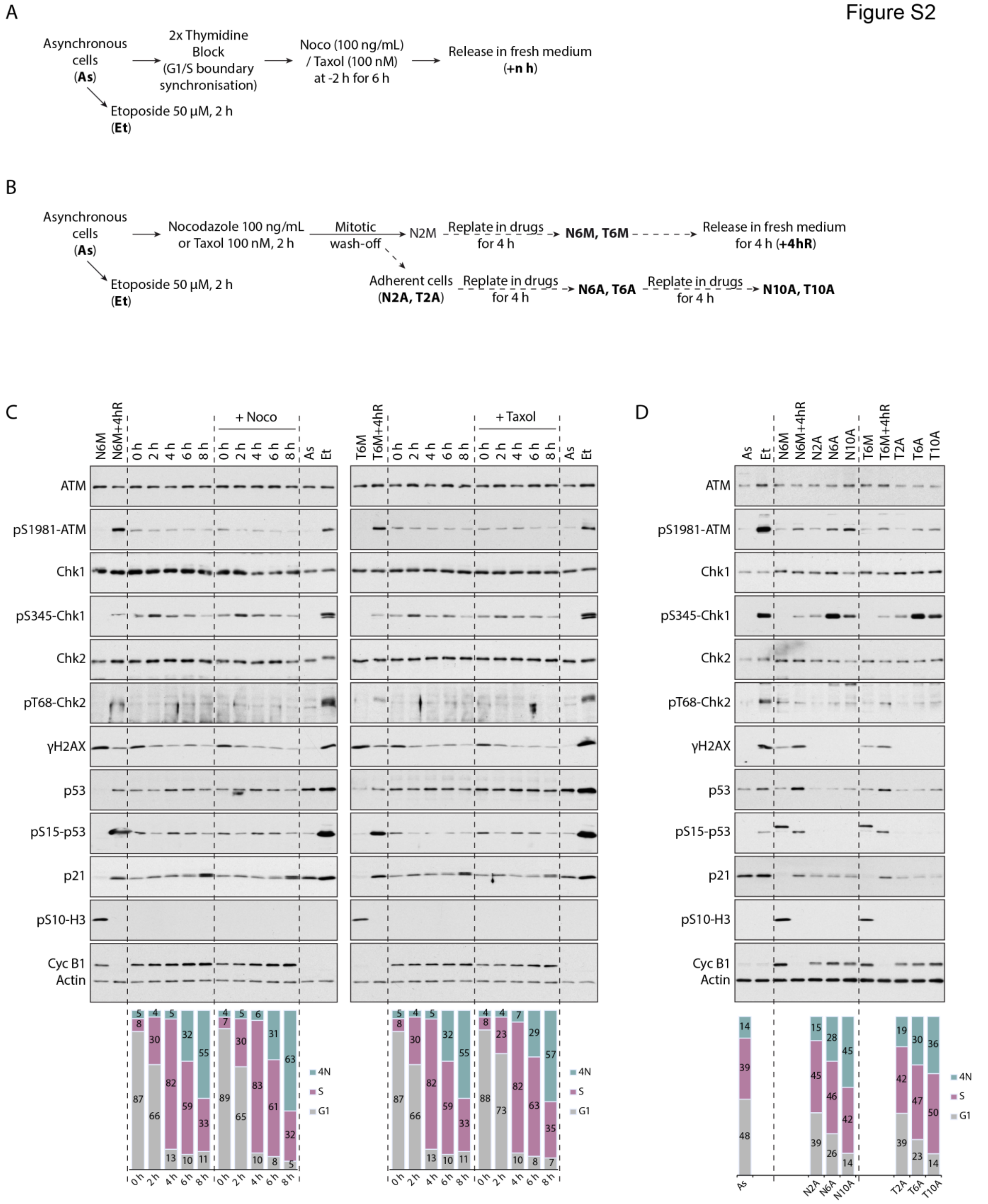

Figure S2. Nocodazole and taxol do not affect DNA damage signalling and cell cycle progression in interphase cells.

A. Protocol for the analysis of effects of nocodazole or taxol on cells synchronised at G1-S boundary and released into the cell cycle. Asynchronous U2OS cells were synchronised at the G1/S boundary by a double thymidine block. 2 h before being released from the second block,

cells were treated with or without nocodazole or taxol for 4 h. Cells were then released with or without the drug and collected every 2 h. Asynchronous (As) and etoposide-treated cells (Et) were also used as controls.

B. Protocol for analysis of interphase cells treated with nocodazole or taxol. U2OS cells were treated with nocodazole or taxol for 2 h, after which time mitotic cells were collected and either replated in drug for a further 4 h (N6M, T6M) or released into fresh media for 4 h (N6M+4hR, T6M+4hR). After wash-off, adherent cells were either collected (N2A, T2A) or incubated for a further 4 h in drug. Adherent cells were obtained after removal of mitotic cells by wash-off (N6A, T6A). This process was repeated to obtain N10A and T10A samples. Asynchronous (As) and etoposide-treated cells (Et) were also used as controls.

C. G1 cells collected as indicated in **A** were analysed by immunoblotting using the specified antibodies (upper). Cell cycle profiles were characterised using propidium iodide staining and flow cytometry (lower).

D. Samples collected as indicated in **B** were analysed by immunoblotting using the specified antibodies (upper). Cell cycle profiles were characterised using propidium iodide staining and flow cytometry (lower).

Figure S3

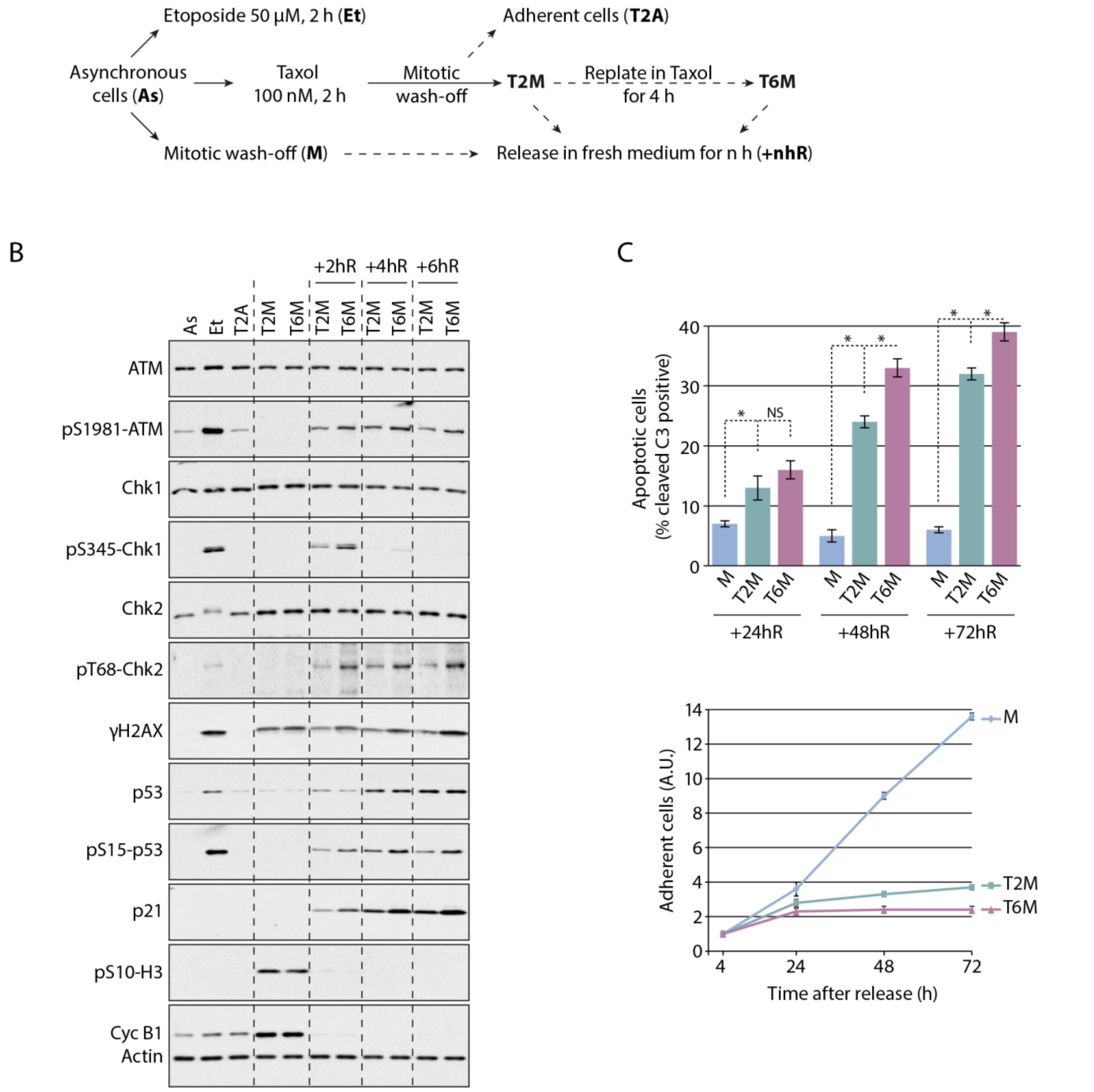

Figure S3. The period of mitotic arrest controls subsequent DNA damage signalling, cell cycle progression and apoptosis.

A. Experimental protocol. Following a pre-synchronisation for 2 h in taxol, mitotic U2OS cells were washed-off (T2M) and either released in fresh media (+nhR) or replated in taxol for further 4 h (T6M) then released for indicated times. After mitotic wash-off, remaining adherent cells (T2A) were collected to control the interphase effects of taxol. Control untreated mitotic cells were also collected by washing-off asynchronous cultures (M). Asynchronous (As) and etoposide-treated cells (Et) were used as controls.

B. The period of mitotic arrest controls the subsequent DNA damage signalling. DNA damage signalling in cells released from various times in mitosis, as depicted in **A**, was analysed by immunoblotting using the specified antibodies.

C, D. The period of mitotic arrest controls the subsequent cell viability and proliferation. U2OS cells treated as described in **A** were collected at indicated times. **(C)** Cells were incubated with a FAM-DEVD-fmk probe and analysed by flow cytometry. The percentages shown represent the amount of active caspase 3/7 positive cells, values are means ± SD ($n \geq 3$). Statistical differences were analysed with the Mann-Whitney test; NS, non-significant, * $p<0.05$. **(D)** The relative amounts of viable, adherent cells were determined by crystal violet assay at indicated times. Values are means ± SD from quadruplicate wells of a representative experiment.

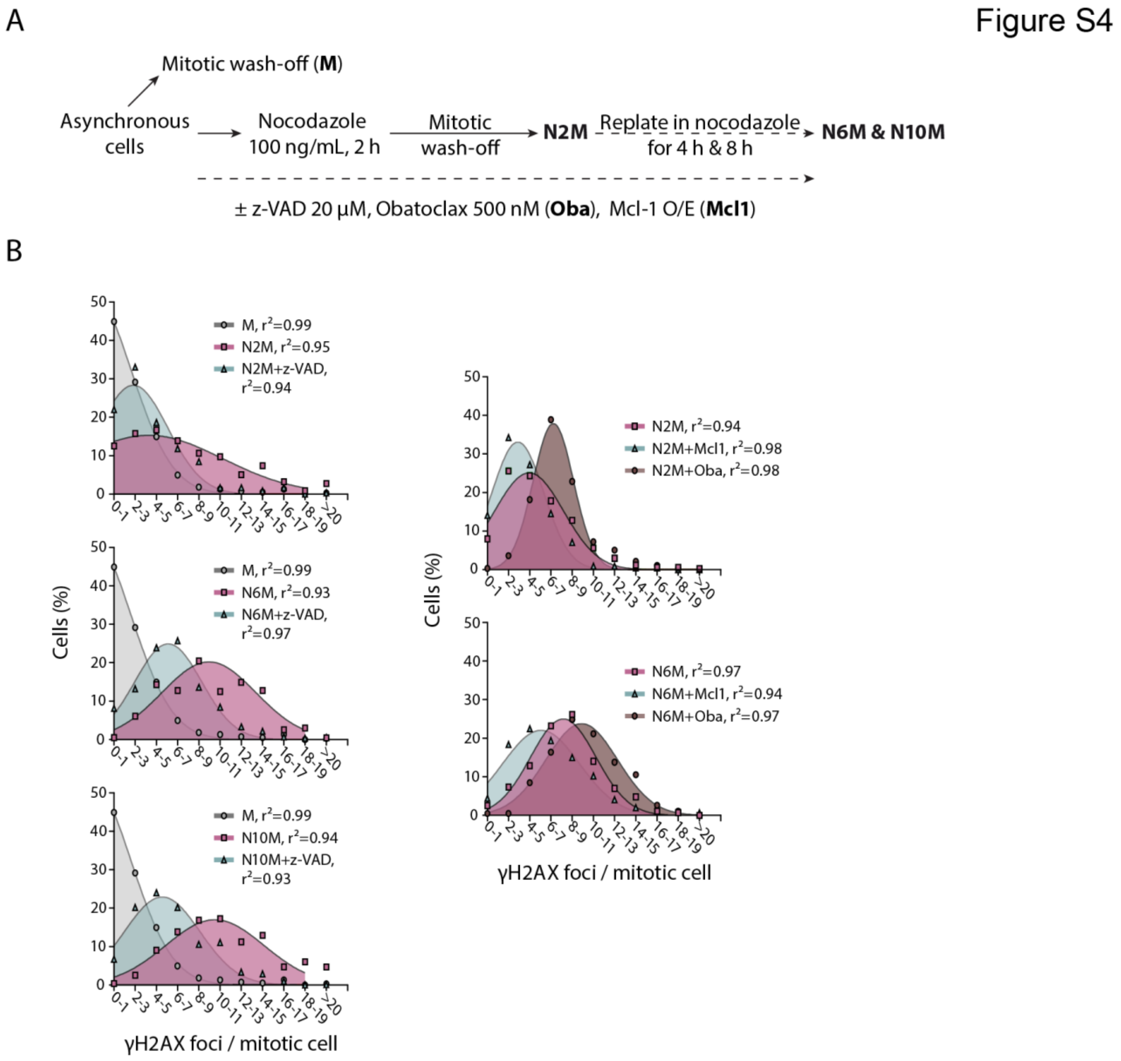

Figure S4. Mitotic arrest elicits a localised caspase-dependent DNA damage response under the control of Bcl-2 family proteins.

A. Following a pre-synchronisation for 2 h in nocodazole (100 ng/mL), mitotic U2OS cells (N2M) were collected and replated in nocodazole for further 4 or 8 h (N6M and N10M). Untreated mitotic cells (M) were collected from asynchronous cultures. Mitosis-arrested cells were also co-treated with the pan-caspase inhibitor z-VAD-fmk (20 µM) or the Mcl-1/Bcl-2/Bcl-x_L inhibitor Obatoclax (500 nM). U2OS cells over-expressing Mcl-1 were prepared by transient transfection. Cells arrested synchronously in mitosis were cytospun and immunostained using a specific anti-γH2AX antibody.

B. Graphs represent the distribution of γH2AX foci counted in at least 200 cells per treatment in a representative experiment. Distributions were fitted with a Gaussian regression.

Figure S5

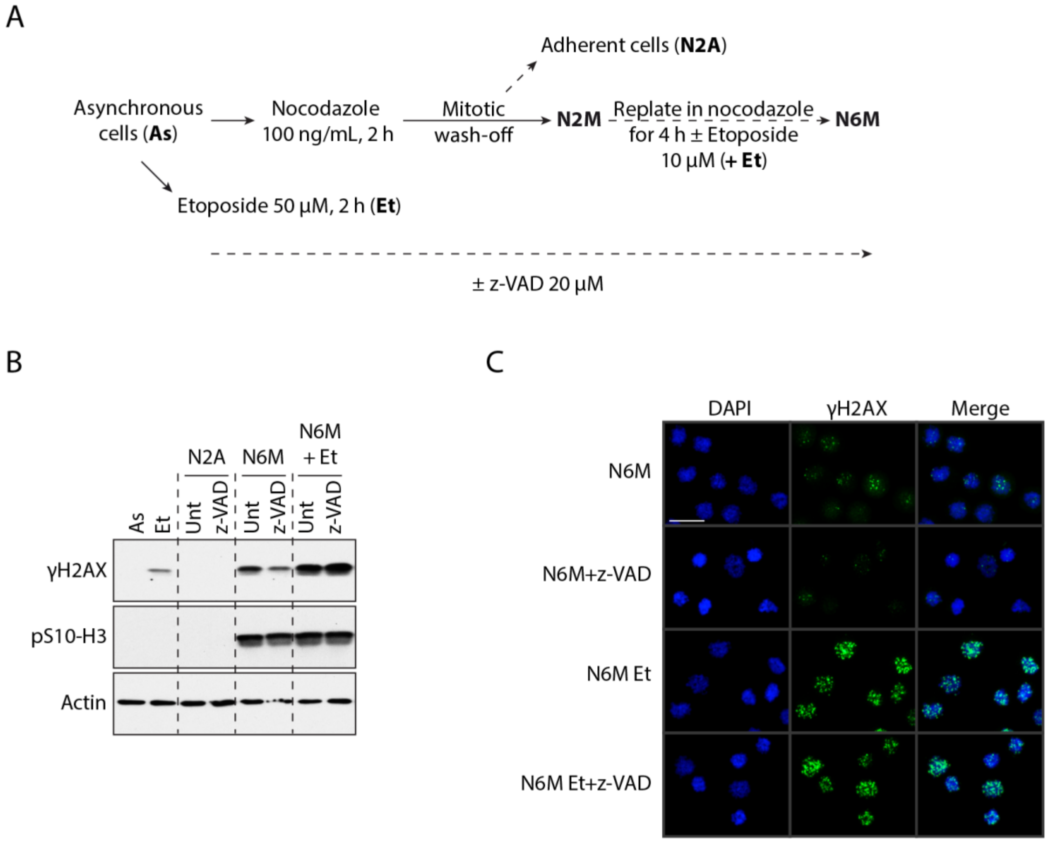

Figure S5. Etoposide-induced DNA damage in mitotically arrested cells is not affected by caspase inhibition.

A. Following a pre-synchronisation for 2 h in nocodazole, mitotic U2OS cells were collected and replated in nocodazole for further 4 h (N6M) with or without z-VAD-fmk and/or etoposide where indicated. After collection of mitotic cells, remaining adherent cells (N2A) were collected to control the interphase effects of nocodazole; asynchronous (As) and etoposide-treated cells (Et) were also used as controls.

B. Cells were treated as indicated in **A** and analysed by immunoblotting using the specified antibodies.

C. Cells treated as indicated in **A** were cytospun and immunostained using a specific anti-γH2AX antibody. Representative microscopic fields are shown; scale bar, 40 µm.

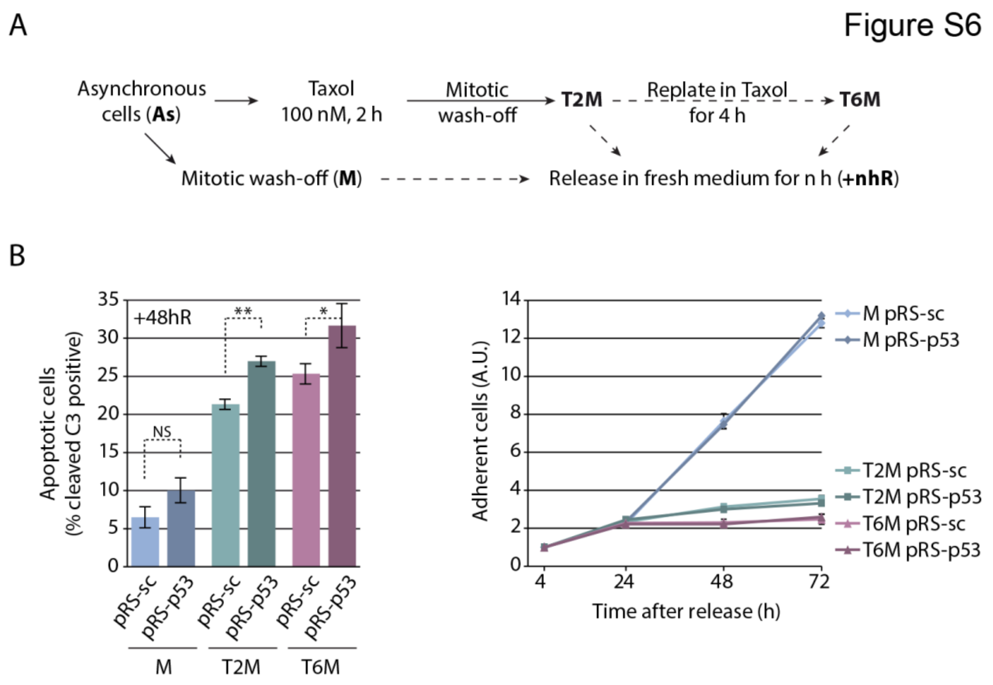

Figure S6. The role of p53 in the fate of cells following a mitotic arrest.

A. Following a pre-synchronisation for 2 h in taxol, U2OS cells in which p53 was stably depleted by shRNA (pRS-p53) and control cells transfected with a scrambled shRNA (pRS-sc), were collected (T2M) and either released in fresh media (+nhR) or replated in taxol for further 4 h (T6M) then released for indicated times. Untreated mitotic cells (M) were also collected from asynchronous cultures.

B. Role of p53 in the viability of cells following a mitotic arrest. Cells collected as indicated in **A** were released in fresh media for 24 to 72 h. On the left, cells were incubated with a FAM-DEVD-fmk probe and analysed by flow cytometry. The percentages shown represent the amount of active caspase 3/7 positive cells, values are means ± SD (n=3). Statistical differences were analysed with the Mann-Whitney test; NS, non-significant, * $p<0.05$, ** $p<0.01$. On the right, the numbers of viable, adherent cells were determined by following cell proliferation using IncuCyte-based cell density analyses. Values are means ± SD from quadruplicate wells of a representative experiment.

Figure S7

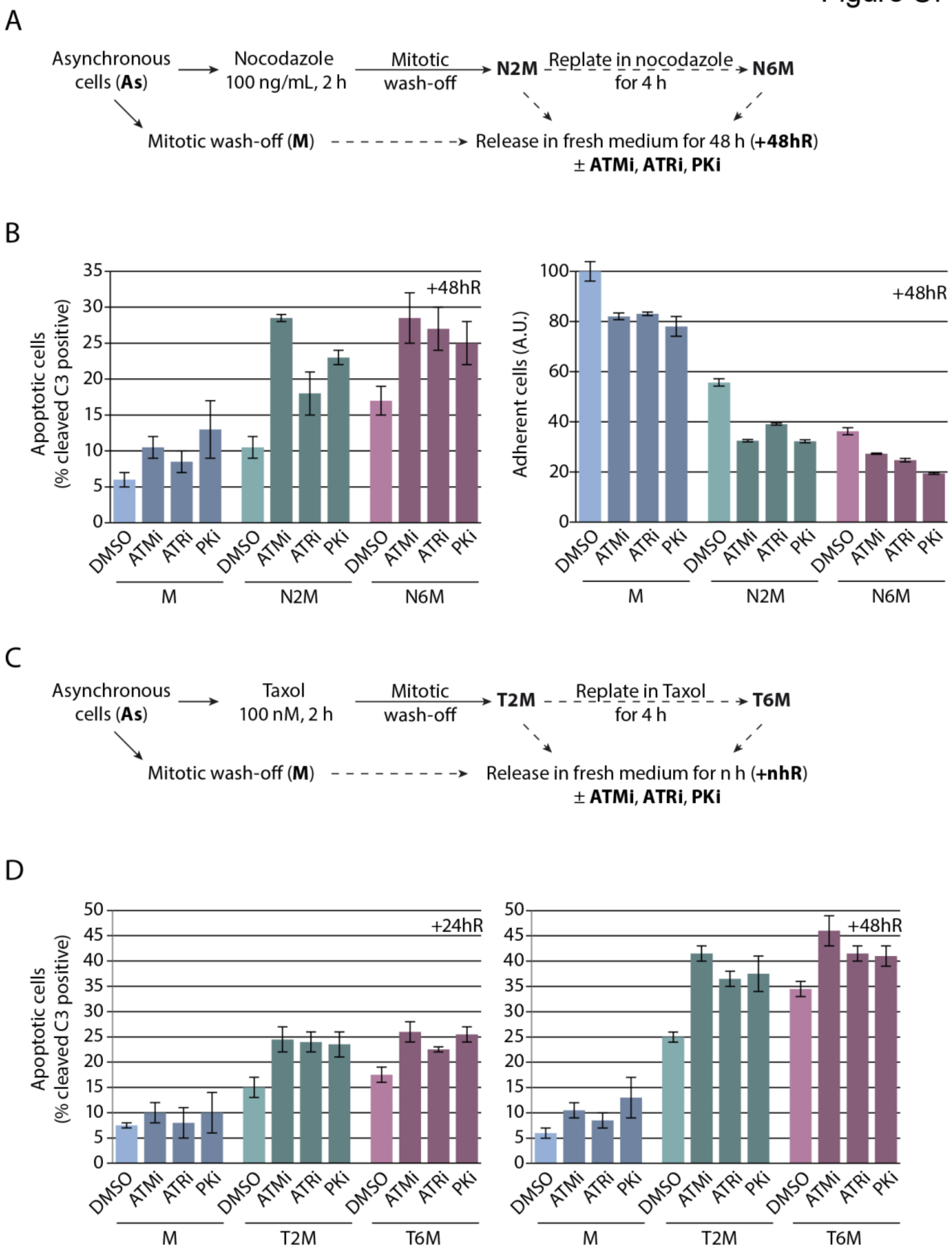

Figure S7. Inhibition of DNA damage response kinases enhances the effects of microtubule poisons.

A. Following a pre-synchronisation for 2 h in nocodazole, mitotic U2OS cells were washed-off (N2M) and either released in fresh media for 48 h or replated in nocodazole for further 4 h (N6M) then released. Control untreated mitotic cells were also collected by washing-off asynchronous cultures (M). Specific inhibitors of ATM (KU55933, 10 µM, ATMi), ATR

(NU6027, 10 μM, ATRi) or DNA-PK (NU7441, 1 μM, PKi) were added at the time of release.

B. Inhibition of the PIKKs reduces the viability of cells following a mitotic arrest. On the left, cells treated as depicted in **A** were incubated with a FAM-DEVD-fmk probe and analysed by flow cytometry. The percentages shown represent the amount of active caspase 3/7 positive cells, values are means ± SD from triplicate wells of a representative experiment. On the right, the relative amounts of viable, adherent cells were determined by crystal violet assay. Values are means ± SD from quadruplicate wells of a representative experiment.

C. Following a pre-synchronisation for 2 h in taxol, mitotic U2OS cells were washed-off (T2M) and either released in fresh media for 24 or 48 h or replated in taxol for further 4 h (T6M) then released. Control untreated mitotic cells were also collected by washing-off asynchronous cultures (M). Specific inhibitors of ATM (KU55933, 10 μM, ATMi), ATR (NU6027, 10 μM, ATRi) or DNA-PK (NU7441, 1 μM, PKi) were added at the time of release.

D. Inhibitions of the PIKKs reduce the viability of cells following a mitotic arrest. Cells treated as indicated in **C** were incubated with a FAM-DEVD-fmk probe and analysed by flow cytometry. The percentages shown represent the amount of active caspase 3/7 positive cells, values are means ± SD from triplicate wells of a representative experiment.

Figure S8

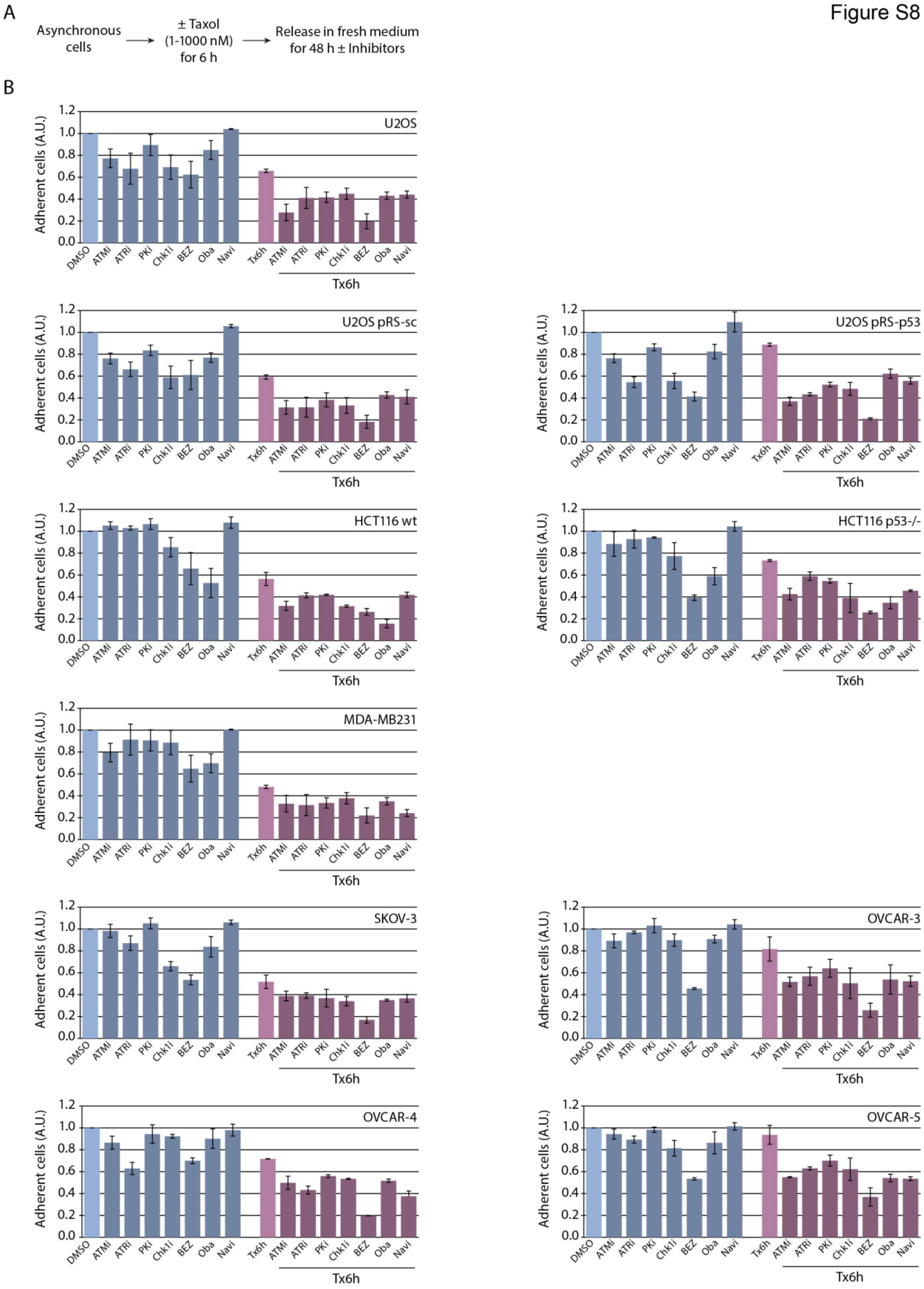

Figure S8. BH3 mimetics and inhibitors of the DNA damage response sensitise cancer cells to taxol.

A. Asynchronous cultures of indicated cell lines were treated with taxol for 6 h then released in fresh media with or without DNA damage signalling kinase inhibitors (ATMi, ATRi, DNA-PKi (PKi), Chk1i, BEZ) and Bcl-2 inhibitors Obatoclax (Oba), Navitoclax (Navi) for 48 h.

B. Indicated cell lines were treated as depicted in **A** with taxol and the different inhibitors. The graphs show the relative amounts of viable, adherent cells determined by crystal violet assay. Values are means ± SD from three independent experiments obtained for 100 nM taxol treatments of U2OS and MDA-MB231 cells and 10 nM treatments of HCT116, SKOV-3 and OVCAR-3, 4, 5 cells. All the cell lines were released in that case in ATMi (10 μM), ATRi (10 μM), PKi (1μM), Chk1i (1 μM), BEZ (50 nM), Oba (50 nM) and Navi (200 nM).

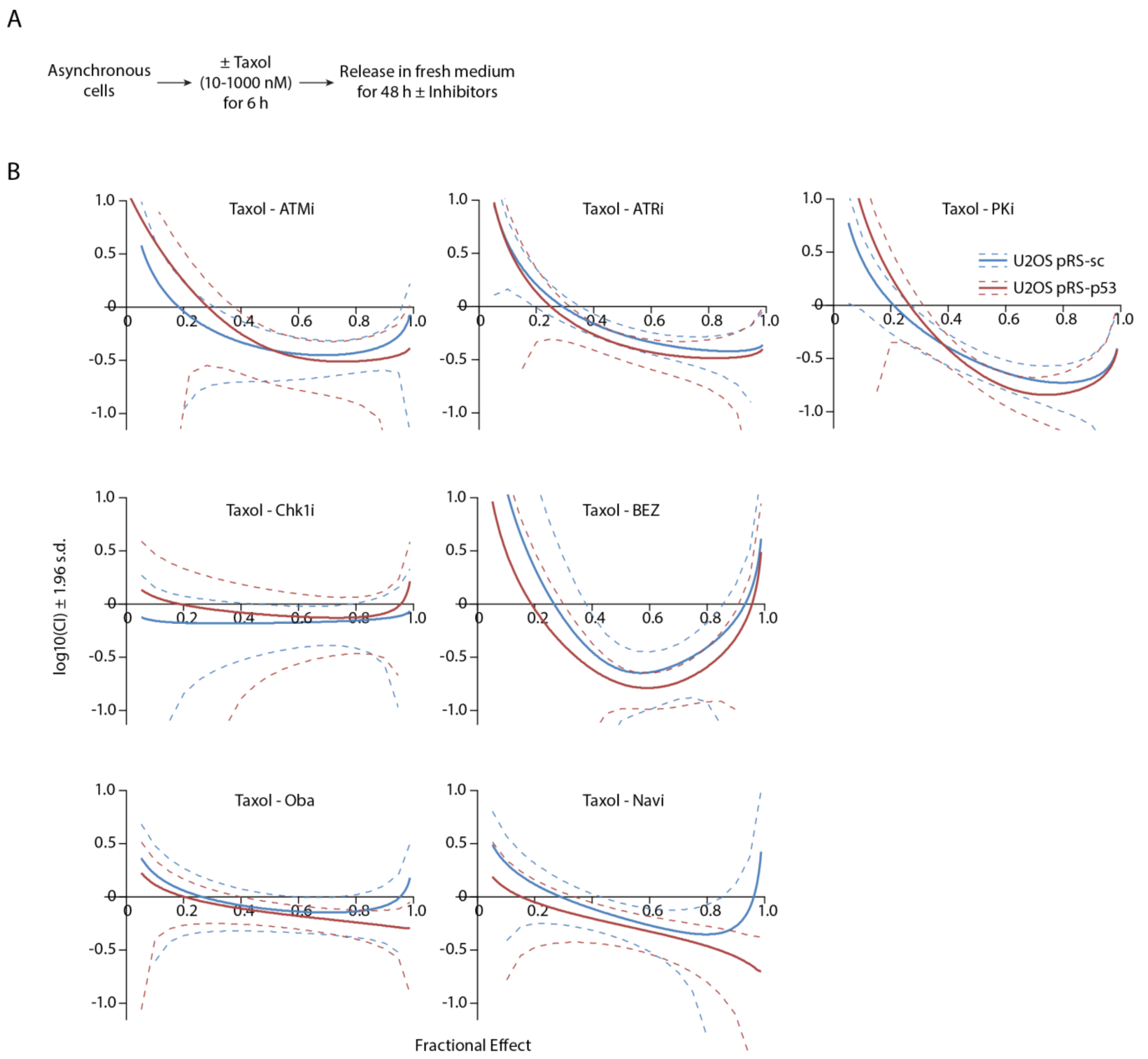

Figure S9. BH3 mimetics and inhibitors of the DNA damage response synergise with taxol in U2OS cells independently of p53.

A. Asynchronous cultures of U2OS cells in which p53 was stably depleted by shRNA (pRS-p53) and control cells transfected with a scrambled shRNA (pRS-sc) were treated with taxol for 6 h then released in fresh media with or without DNA damage signalling kinase inhibitors ATMi (1-100 µM), ATRi (1-100 µM), DNA-PKi (PKi, 0.1-10 µM), Chk1i (0.1-10 µM), BEZ (5-500 nM) or BH3 mimetics Obatoclax (Oba, 5-500 nM) or Navitoclax (Navi, 20- 2000 nM) for 48 h.

B. Combination indexes (CI) obtained by treating U2OS cells with taxol in combination with DNA damage signalling inhibitors and BH3 mimetics. Cells were treated as indicated in A and the relative amount of live adherent cells was quantified by the crystal violet assay. The software Calcusyn was used to compute the data obtained by treating cells with at least four

different concentrations of taxol and of the inhibitors at constant ratios resulting in Fractional Effect / $\log_{10}$(CI) plots as shown in a representative experiment, according to Chou [20]. CI (solid lines) are displayed ± 1.96 SD (dashed lines) evaluated by Calcusyn algebraic estimates. The log10(CI) values obtained at given concentrations depict an antagonism between the drugs when >0, an additive effect when equal to 0 and a synergism when <0.
